# Leukocyte dynamics in *Cynomolgus* monkeys following heterotopic heart allotransplantation under costimulation pathway blockade

**DOI:** 10.3389/fimmu.2025.1664463

**Published:** 2025-10-10

**Authors:** Gheorghe Braileanu, Agnes M. Azimzadeh, Tianshu Zhang, Lars Burdorf, Richard N. Pierson III

**Affiliations:** 1 Department of Surgery, University of Maryland Medical Center, Baltimore, MD, United States; 2 Division of Cardiac Surgery, Department of Surgery and Center for Transplantation Science, Massachusetts General Hospital and Harvard Medical School, Boston, MA, United States

**Keywords:** leucocyte dynamics, cynomolgus macaque, heart heterotopic allotransplantation, intragraft lymphocytes, T cells subpopulation

## Abstract

**Rationale:**

It was hypothesized that the dynamics of leukocyte populations in peripheral blood (PB) or peri-graft lymph nodes (LNs) in cynomolgus monkey recipients of a heterotopic heart allotransplant, completed with the determination of graft-infiltrating lymphocyte (GIL) populations at explant, may vary in association with immune rejection mechanisms or immunomodulatory treatments.

**Methods:**

Among 15 cynomolgus monkey recipients of heterotopic heart allografts, 13 were treated with a variety of costimulation-blocking immunosuppressive (IS) agents targeting CD80/CD86, CD28, CD40, or CD154, and two were untreated (controls). Leukocyte populations were characterized using hemocytometry and flow cytometry.

**Results:**

In PB, neutrophils and monocytes increased significantly (p < 0.001) during the first 2 weeks after transplant. Eosinophils and monocytes steadily increased after transplant, peaking around the time of graft failure (p < 0.01), a trend most prominent in association with belatacept. After the initial nadir on day 1 after transplant, PB lymphocytes increased steadily, particularly in association with belatacept and hu5c8, to a peak 1 week before graft rejection (p < 0.05), like CD3 cells. In PB, the CD4/CD8 ratio consistently trended down in all treated groups, most prominently in association with 5c8. In LNs at explant, CD4 cells outnumbered CD8 cells (p < 0.001), whereas in graft-infiltrating lymphocytes (GILs), CD8 cells predominated (p < 0.001). Among GILs at the time of rejection, CD8+CD62− central and effector memory cells were prominent, along with CD4+CD8+ T cells and IgD−CD27− B cells. At explant time, analysis of CD3 CD127lowCD25highFoxp3+ cell populations identified in GILs two clusters of CD4+CD8+ and three clusters of CD8 cells, which were expanded relative to PB or LNs.

**Conclusions:**

Observations regarding CD8 T-cell subpopulations in PB, LNs, and GILs support the conventional paradigm regarding their role as key effector cells mediating graft injury. The prominence of CD4+CD8+CD127lowCD25highFoxp3+ T cells and that of IgD−CD27− B cells among GILs have not previously been described. Expansion of circulating eosinophils around the time of rejection may implicate these cells in rejection mechanisms. Comparison of graft lymphocyte subpopulations with LNs or PB highlights mechanistically plausible differences that justify further efforts to elucidate their roles in graft injury and protection as a strategy to identify new candidate approaches to prevent rejection and promote tolerance.

## 1 Introduction

Cynomolgus monkey models are useful in predicting how various monoclonal antibodies that block different costimulation pathways may influence the duration of organ allograft survival in humans and interrogating related immune mechanisms (1–3). Without intervention, a heterotopic heart allotransplant is rejected after approximately 7 days in monkeys, whereas in association with various costimulatory pathway-blocking reagents under study by our group, graft survival was consistently prolonged beyond 90 days in most recipients. Leukocyte fluctuations over time reflect the adaptive reactions of the physiological immune mechanisms to the effects of early proinflammatory stress induced by the surgical procedure of transplantation and the responses to perceived immune “threat” posed by continuous exposure to graft antigens, superimposed on the consequences of the different costimulation-blocking immunosuppressive (IS) treatments. The dynamics of peripheral blood leukocytes and the modulation of lymphocyte subpopulations in lymph nodes (LNs) or GILs in association with IS antibody treatments (1, 2, 4–6) after heart heterotopic allograft transplantation have not been previously reported in-depth.

We hypothesized that a pilot investigation of the numbers and phenotypes of peripheral blood (PB) leukocyte populations over time after transplantation in animals with stable graft function on IS treatments up to around the time of graft rejection may yield new insights into immune mechanisms of rejection and differential effects of various costimulation pathway-blocking agents under study. We speculated that comparing the phenotypes of PB lymphocytes and graft-adjacent mesenteric LNs around the time of the transplant and the time of graft rejection with GILs at the time of explant may generate testable hypotheses regarding how to enhance the positive effects of costimulation pathway-blocking treatments and perhaps contribute to clinical applications for future heart transplant recipients.

## 2 Materials and methods

### 2.1 Study design

From our allotransplant experiments performed between 2008 and 2016, we identified 13 monkeys treated with various combinations of different costimulation pathway-blocking treatments in which graft survival was prolonged beyond 80 days (defined as “effective” treatment) and recovered after the removal of their grafts due to rejection following treatment cessation, allowing post-rejection monitoring (**Table 1**). Subjects received one of four “platform” CD40/CD154- or CD28/B7-directed costimulation pathway-blocking antibody treatments: anti-CD40 (2C10R4) [Non-Human Primate Reagent Resource Center (NHPRC, Boston, MA, USA), lot 020112G; n = 3]; anti-CD154 (hu5c8), a “rhesus-ized” mouse-anti-human 5c8 IgG1k (NHPRC, lot LH16-08; n = 3); anti-CD28 (FR104), a PEGylated Fab that selectively blocks CD28 without T-cell activation (lot P from Dr Bernard Vanhove, INSERM U643, Nantes, France; n = 4); or a CTLA4-Ig Fc construct, which binds to and blocks CD80 and CD86 (belatacept, Bristol Myers Squibb, New York, NY, USA; n = 3). Of the treated monkeys, six received one supplementary treatment as detailed in **Table 1**, including anti-CD20 (Rituxan, Genentech, Inc., South San Francisco, CA, USA) (n = 1), anti-ICOSL (ICOS IgG) (lot 072710 from Dr. Mohamed Sayegh, Harvard Medical School, Boston, MA, USA) (n = 1), ethylene carbodiimide (ECDI)-treated donor splenic antigen-presenting cells (APCs; n = 2), anti-CD40 (2C10R4, n = 1), or anti-CD154 (hu5c8, n = 1) in addition to FR104. For analysis of the results, the treated monkeys were divided into four major costimulation blockade groups (**Table 1**). Heart allografts were explanted based on rejection criteria described below, except for two well-functioning grafts that were explanted on day 90 in the 2C10R4 group by the protocol in place at the time these transplants were performed. The two animals that received no immunomodulatory treatment (controls) had the same monitoring regimen as costimulation-treated subjects. In summary, for this retrospective analysis, a total of 15 cynomolgus monkeys (*Macaca fascicularis*) of Indonesian or Chinese origin (Alpha Genesis, Yemassee, SC, USA) were evaluated, for which a comprehensive data set for blood, LNs, and GILs (as detailed below) was available. All experimental procedures were approved by the Institutional Animal Care and Use Committee of the University of Maryland School of Medicine and were conducted in compliance with criteria outlined in the Guide for the Care and Use of Laboratory Animals published by the National Institutes of Health.

**Table 1:** Monkey demographics, treatment groups, and graft outcomes.

Monkey	Origin	Sex	Age (years)	Weight (kg)	Treatment groups	Treatment details	Graft survival (days)	ABO
1	Chinese	M	6.1	6.3	2C10R4		90*	AB
2	Indonesian	M	6.3	5.1	2C10R4	+ aCD20	90*	A
3	Indonesian	M	5.4	5.0	2C10R4	+ ICOS Ig	119	A
4	Chinese	F	7.2	5.3	hu5c8		174	B
5	Indonesian	M	5.4	3.7	hu5c8	+ ECDI	135	A
6	Chinese	M	4.8	4.5	hu5c8	+ ECDI + lateFR104	114	AB
7	Chinese	M	6.4	10.3	Belatacept	10 mg/kg	121	AB
8	Chinese	M	6.3	6.4	Belatacept	10 mg/kg	124	AB
9	Indonesian	M	7.6	5.8	Belatacept	5 mg/kg	124	A
10	Chinese	M	5.5	8.9	FR104		82	AB
11	Chinese	M	5.8	6.4	FR104		184	B
12	Chinese	M	5.3	7.8	FR104	+2C10R4	172	AB
13	Chinese	M	5.0	9.8	FR104	+ hu5c8	176	B
14	Chinese	M	7.7	12.7	No Rx		7	AB
15	Chinese	M	5.3	12.6	No Rx		7	A

2C10R4, anti-CD40; hu5c8, anti-CD154; belatacept, a fusion protein between the Fc fragment of human IgG1 immunoglobulin and the extracellular domain of CTLA-4; FR104, anti-CD28; ECDI, ethylene carbodiimide-treated donor splenic antigen-presenting cells.

*Beating graft explanted with the study protocol in place at that time.

### 2.2 Intra-abdominal heterotopic cardiac allotransplantation

The cardiac allotransplantation procedure was previously reported (1, 3). Allotransplant heart monitoring and criteria for allograft explant are summarized in **Supplementary Information** (**SI-1**).

### 2.3 Treatment regimens

2C10R4 was administered in a dose of 30 mg/kg on post-operative days (PODs) 0, 3, 7, and 14; 10 mg/kg on PODs 21, 28, 35, and 42; and 20 mg/kg on PODs 56 and 84 to three recipients. Two of these animals received additional peri-transplant treatment with anti-CD20 (20 mg/kg on PODs −1, 7, 14, and 21; n = 1) or ICOS-Ig (5 mg/kg on PODs 63, 70, 77, 84, 91, 98, and 105; n = 1). hu5c8 was administered to three animals at 30 mg/kg on PODs 0, 3, 7, and 14; 10 mg/kg on PODs 21, 28, 35, and 42; and 20 mg/kg on PODs 56 and 84. Two hu5c8-treated animals also received 300 million ECDI-treated donor splenic antigen-presenting cells on the day of transplant and on POD 4. Belatacept was administered in a dose of 5 (n = 1) or 10 (n = 2) mg/kg on PODs 0, 4, 7, 14, 21, 28, 42, 56, 70, and 84. FR104 was administered in a dose of 5 mg/kg on PODs 0, 4, 7, 14, 21, 28, 42, 56, 70, and 84. Two of the FR104-treated animals were additionally treated with either hu5c8 or 2C10R4 and dosed until D84 as the monotherapy group; for analysis purposes, these two animals were included in the FR104-treated group.

### 2.4 Mesenteric lymph node biopsies

Lymph nodes from the mesentery near the allograft were excised on the day (D) of transplant (D0) and on the day of graft explant. The harvested LNs, after weighing, were passed through a 35-μm stainless steel mesh. Collected cells were counted and characterized by flow cytometry.

### 2.5 Graft-infiltrating lymphocytes

A 20-g sample from the explanted heart was used for the isolation of GILs using the method described in SI-2. The isolated lymphocytes were counted and characterized by flow cytometry.

### 2.6 Characterization of leukocytes in peripheral blood

Leukocytes from PB samples collected in Ethylenediaminetetraacetic acid (EDTA) were analyzed at baseline (BL) before transplant and at PODs 1, 7, 14, 21, 21, 28, 43, 49, and 56 following transplantation (“post-transplant interval”). Leukocytes analyzed every 1–2 weeks thereafter were retrospectively evaluated for D−20–24, D−5–10, EXPLANT, D + 7, D + 14-21, and D + 28-35 to graft explant in the setting of rejection (n = 11) or the time of graft explant by protocol (n = 2) (“peri-rejection interval”).

The leukocyte methods used are detailed in SI-3.

The results are presented as data for individual monkeys, as means for each of four major treatment categories, and as means for all treated subjects.

### 2.7 Flow cytometry

For each time point and sample type (blood, LNs, and GILs), 34 fluorescence-conjugated antibodies were organized in 10 flow panels as described in SI-3.

To facilitate meaningful comparisons of different lymphocyte subpopulations between time points or locations (PB, LNs, or GILs), the results of each sample were obtained from the same number of 10,000 CD3 cells or 1,000 B lymphocytes obtained using the weighted downsizing software tool in FCS Express-7 (SI-4).

Cluster analysis of CD3 CD127lowCD25highFoxp3+ cell populations was performed using the k-means methodology of the FCS Express-7 software, starting with the same initial number of CD3 cells for each sample (SI-4, step 7).

### 2.8 Statistical analysis

For each parameter, statistical differences between time points relative to BL, or between locations at explant, were analyzed using a two-tailed t-test. The results were considered significant when p < 0.05. Data are presented as mean ± SEM (error bars).

## 3 Results

### 3.1 Leukocyte dynamics in PB

**Figure 1:**
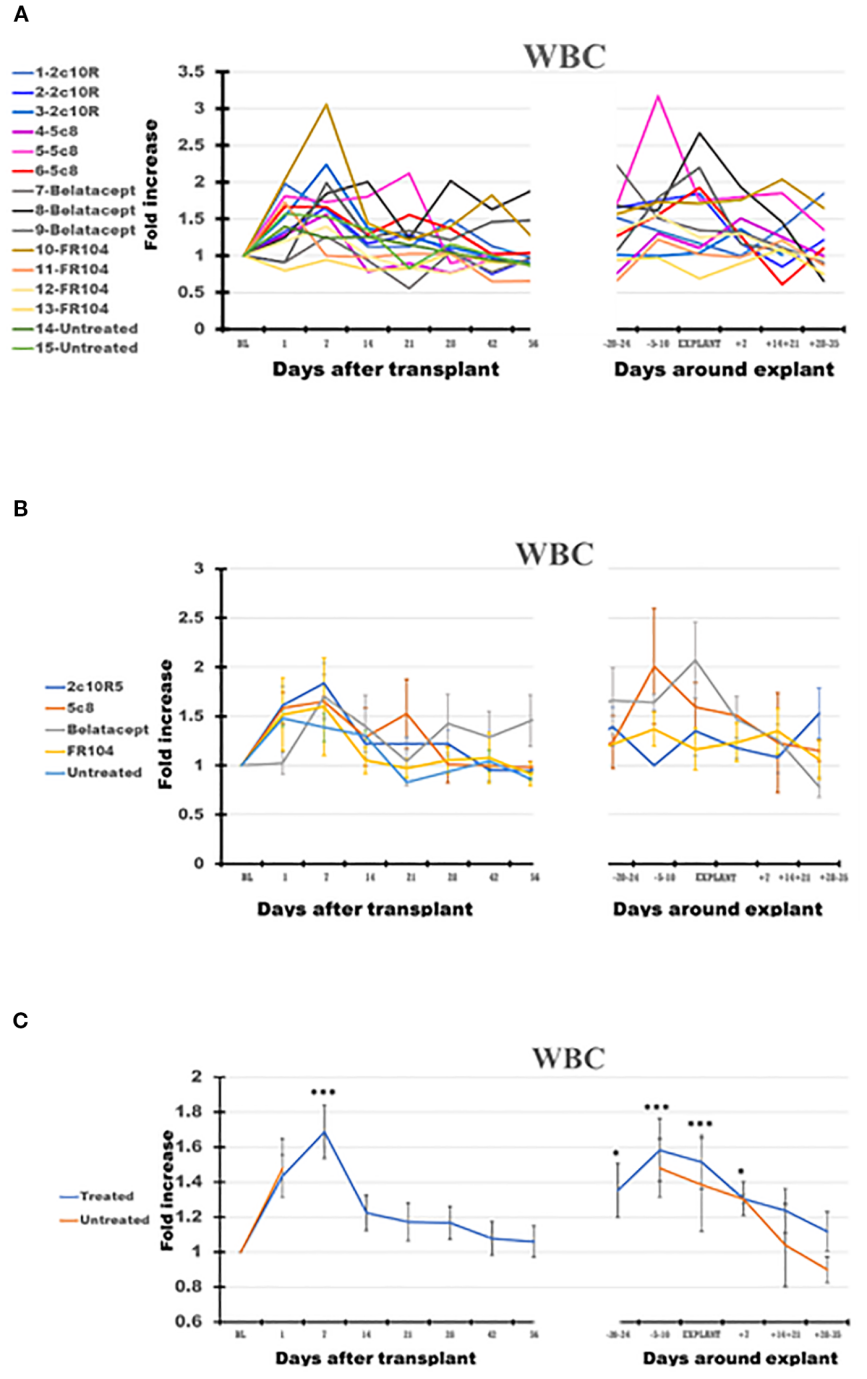
The dynamics of white blood cells (WBC) in peripheral blood (PB) of the cynomolgus monkeys after a heterotopic (abdominal) heart transplantation. Absolute numbers of WBC, measured using the TruCount method (as described in the Materials and Methods section), are represented as fold changes versus values recorded before the transplant [baseline (BL) = mean value presented in **Table 2**]. For each graph, the measurements were represented post-transplant (left panel of each graph) or peri-rejection (right panel of each graph) at the indicated time points. **(A)** Results are shown for each monkey (n = 15) and color-coded as blue (2C10R4), red orange (5c8), gray (belatacept), yellow (FR104), and green spectrum (untreated). **(B)** Results are grouped by treatments, retaining color scheme from panel **(A)** Error bars reflect SEM. **(C)** Mean of all treated monkeys in association with stable graft function for all treatment groups (blue) versus untreated with acute rejection at D7 (orange). For aggregated data, significant differences from BL are indicated for p < 0.05 (*), p < 0.01 (**), and p < 0.001 (***).

White blood cells (WBC) (**Figures 1A–C**) increased significantly in PB after transplantation (p < 0.001 at day +7). For the remainder of the post-transplant interval, during ongoing immunosuppressive treatment, WBC counts generally decreased toward BL by D56. Compared with BL, WBC significantly increased peri-rejection from 1.38-fold on D−20–24 (p < 0.05) to 1.59-fold on D−5–10 before rejection (p < 0.001) and 1.47 at explant (p < 0.001). After graft explant, WBC counts tended to decrease from the peri-rejection peak but were still elevated compared with BL (1.3-fold; p < 0.05) on D + 7 after explant.

**Figure 2:**
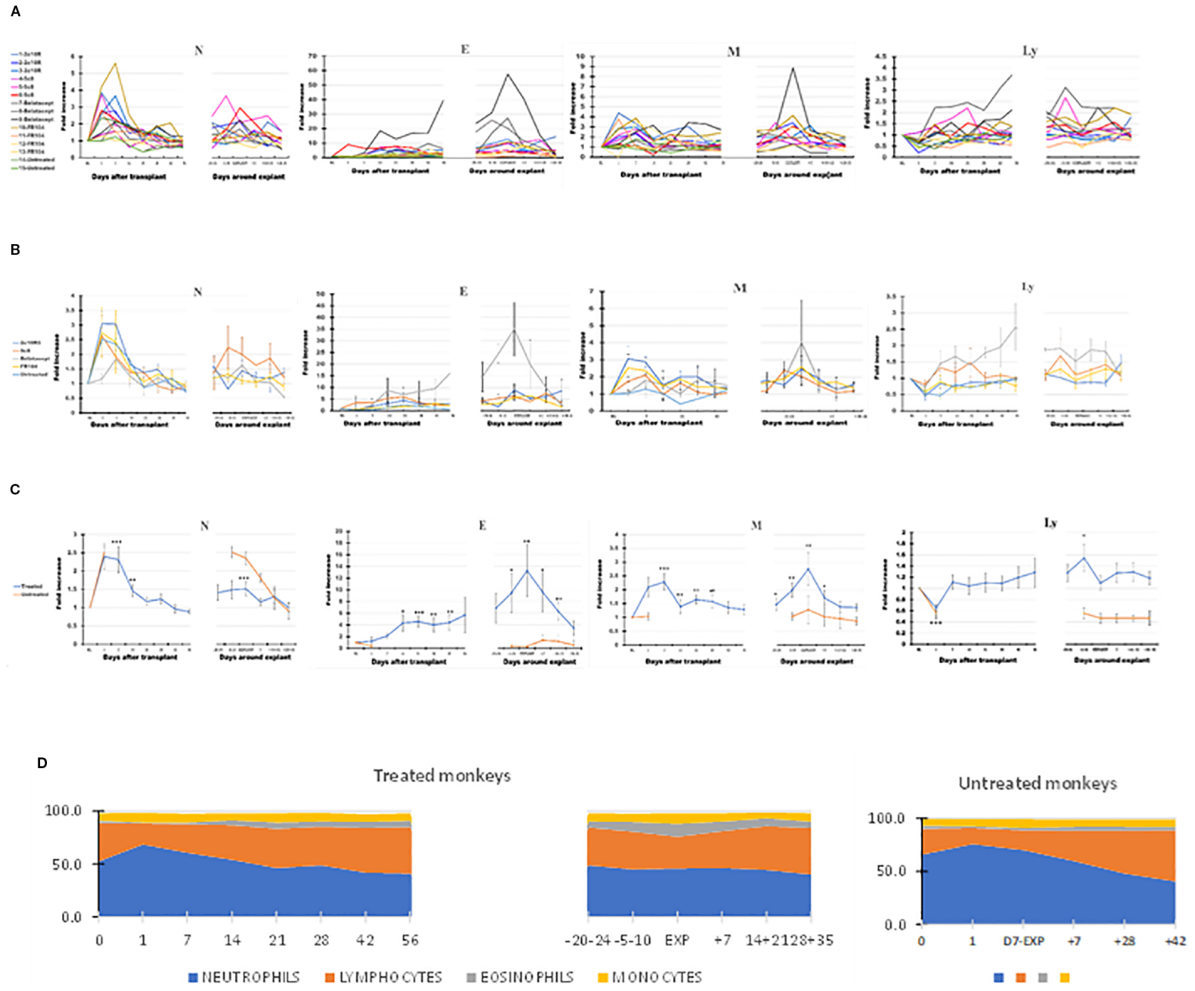
The dynamics of different leukocyte types in peripheral blood (PB) of the cynomolgus monkeys after a heterotopic (abdominal) heart transplantation. Absolute numbers of neutrophils (N), eosinophils (E), monocytes (M), and lymphocytes (Ly), measured using TruCount method (as described in Materials and Methods section), are represented as fold changes versus values recorded before the transplant [baseline (BL) = mean values presented in **Table 2**]. For each graph, the measurements were represented post-transplant (left panel of each graph) or peri-rejection (right panel of each graph) at the indicated time points. **(A)** Results are shown for each monkey (n = 15) and color-coded as blue (2C10R4), red orange (5c8), gray (belatacept), yellow (FR104), and green spectrum (untreated). **(B)** Results are grouped by treatments, retaining color scheme from panel **(A)** Error bars reflect SEM. **(C)** Mean of all treated monkeys in association with stable graft function for all treatment groups (blue) versus untreated with acute rejection at D7 (orange). Neutrophils and monocytes increased significantly in PB during the first 2 weeks after transplant, whereas circulating lymphocytes were reduced during the first week. Eosinophils and monocytes steadily increased and spiked around the time of graft failure in all groups, a trend most prominent in association with belatacept. Circulating lymphocytes increased during the week prior to graft failure. **(D)** Combined dynamic changes in the percentages of neutrophils (blue), lymphocytes (orange), eosinophils (gray), and monocytes (yellow) in treated monkeys (left side of the panel) compared with untreated ones (right side of the panel). For aggregated data, significant differences from BL are indicated for p < 0.05 (*), p < 0.01 (**), and p < 0.001 (***).

Neutrophils (N) and monocytes (M) predominantly account for the initial leukocytosis following transplant. N, eosinophils (E), M, and lymphocytes (Ly) all increased peri-rejection (**Figures 2A–D**, **Table 2**). In all treatment groups, eosinophils in PB increased around the time of graft rejection (**Figure 2B**, panel 2), most notably with belatacept. The relative increase in eosinophils through the post-transplant and peri-rejection intervals reached a peak of 13.3 times higher than BL on average (p < 0.01) at the time of explant (**Figure 2C**, panel 2) before subsiding after graft explant.

**Table 2:** Changes in the numbers of leukocyte subpopulations in PB on the day of cardiac allograft explant (EXPL) relative to baseline (BL).

Leukocyte	BL (cell count/μL)	EXPL (fold increase)	Significance (p < 0.05)
WBC	11,149 ± 1,043	1.51	+
Neutrophils	5,365 ± 685	1.50	+
Lymphocytes	4,585 ± 508	1.09	−
Monocytes	797 ± 144	2.75	+
Eosinophils	259 ± 64	13.30	+
CD3	3,259 ± 320	1.05	−
NK	188 ± 90	1.38	−
B cells	1,290 ± 199	1.01	−

PB, peripheral blood; WBC, white blood cells.

In contrast to N, E, or M, Ly decreased significantly in the first week after transplant and then increased gradually, trending above baseline levels during the second or third month after transplant and becoming significantly elevated in the week preceding explant (days 5–10; p < 0.05 relative to BL). More prominent PB Ly expansion was observed in association with 5c8 and belatacept treatments relative to 2C10R4 or FR104 treatment (**Figure 2B**, panel 4). Compared with the pre-explant peak in PB Ly, explant was associated with a decrease in the absolute number of circulating lymphocytes (**Figure 2C**, panel 4). In post-explant follow-up, PB Ly counts were not significantly different compared with BL.

**Figure 3:**
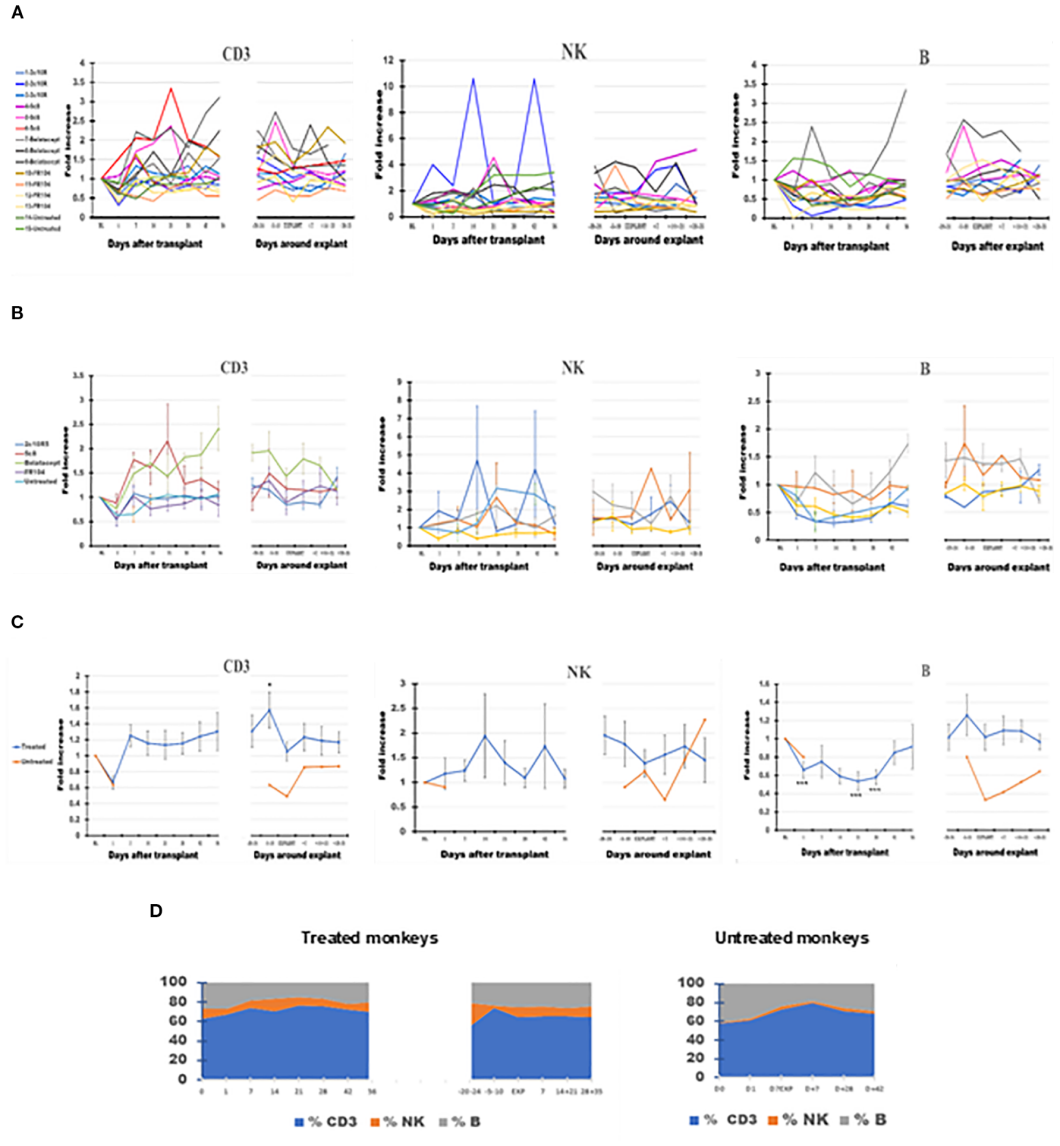
The dynamics of absolute lymphocyte numbers in peripheral blood (PB) of the cynomolgus monkeys after a heterotopic (abdominal) heart transplantation. Absolute numbers of CD3+ T cells (CD3), NK cells (NK), and B cells (B), measured based on TruCount method results for lymphocytes (Ly) (as described in Materials and Methods section) as well as on Ly subset percentages defined by flow cytometry (SI 3 and 4), are represented as fold changes versus values recorded before the transplant [baseline (BL) = mean values presented in **Table 2**]. For each graph, the measurements were represented post-transplant (left panel of each graph) or peri-rejection (right panel of each graph) at the indicated time points. **(A)** Results are shown for each monkey (n = 15) and color-coded as blue (2C10R4), red orange (5c8), gray (belatacept), yellow (FR104), and green spectrum (untreated). **(B)** Results are grouped by treatments, retaining color scheme from panel **(A)** Error bars reflect SEM. **(C)** Mean of all treated monkeys in association with stable graft function for all treatment groups (blue) versus untreated with acute rejection at D7 (orange). After a decrease on D + 1 after transplant, CD3+ T cells tended to expand in association with 5c8 and belatacept treatments relative to other groups in the first 3 weeks after transplant; the relative increase persisted only in belatacept group through 8 weeks after transplant. Except for an increase in circulating CD3 T cells approximately 1 week before graft failure, no significant changes were observed in peripheral lymphocyte phenotype around the time of graft rejection. **(D)** Combined dynamic changes in the percentages of CD3 cells (blue), NK cells (orange), and B cells (gray) in treated monkeys (left side of the panel) compared with untreated ones (right side of the panel). For aggregated data, significant differences from BL are indicated for p < 0.05 (*), p < 0.01 (**), and p < 0.001 (***).

In the two untreated monkeys that rejected their graft at POD + 7, WBC increased on POD + 1, decreased slightly on the day of explant, and thereafter generally decreased until day 35 after explant (**Figure 1C**), a pattern similar to that of treated animals. Neutrophils remained elevated at the time of rejection on POD7 before decreasing to baseline levels by 14–21 days post-explant (**Figure 2C**, panel 1). Without treatment, PB Ly numbers decreased on the first day after transplant and remained lower compared with those of treated monkeys through day 35 after explant (**Figure 2C**, panel 4); both CD3 T cells and CD20 B cells remained lower during post-rejection post-explant follow-up in untreated monkeys compared with treated recipients (**Figure 3C**, panels 1 and 3). The relative expansion of E or M at explant in treated monkeys was not seen in untreated monkeys (**Figure 2C**, panels 2 and 3).

The various costimulation pathway-blocking treatments did not exhibit significantly different patterns in the dynamics of leukocyte subpopulations in the post-transplant or peri-rejection intervals, although we did observe differences in amplitude. For instance, eosinophilia was particularly noticeable in all three belatacept-treated animals in anticipation of graft failure and at explant (**Figure 2A**, panel 2); in addition, the numbers of PB Ly tended to be higher in the belatacept-treated group (**Figure 2B**, panel 4).

### 3.2 Lymphocyte subset dynamics in peripheral blood

Compared with BL, the number of CD3+ T cells briefly fell on D + 1 before increasing by D7 and generally remained elevated during post-transplant follow-up, reaching the peak 1 week before explant (p < 0.05) (**Figures 3A–D**). CD3 then decreased toward BL on the day of explant before trending up again during the post-explant follow-up (**Figure 3C**, panel 1). Anti-CD154- and anti-CD28-based treatments tended to be associated with less expansion of CD3 cells relative to anti-CD40 or belatacept-treated monkeys during the post-transplant phase (**Figure 3B**, panel 1). For belatacept treatment, CD3 T-cell numbers continued to be higher around the time of graft failure relative to the other treatment groups or untreated monkeys (**Figure 3B**, panel 1).

B-cell numbers also decreased at D + 1 (p < 0.001) but, unlike CD3 cells, remained at a low level for the first month after transplant before recovering to near BL around D41 (**Figure 3C**, panel 3). B cells also tended to peak during the week before explant and then decreased toward BL during post-explant convalescence. With belatacept- and hu5c8-based treatments, B-cell numbers tended to be higher compared to those of other treatment groups (**Figure 3B**, panel 3). For other treatment groups, overall B-cell numbers did not deviate significantly from BL around the time of graft rejection.

Driven largely by variations in one 2C10R4-treated animal and one belatacept-treated animal, NK cells appeared to be significantly increased (p < 0.05) compared with BL at D−20 before explant (**Figures 3A–C**, panel 2). The trend toward expanded NK cells in PB throughout the remainder of the study was otherwise not statistically significant in any group.

### 3.3 CD3+ T-cell subset dynamics in PB

**Figure 4:**
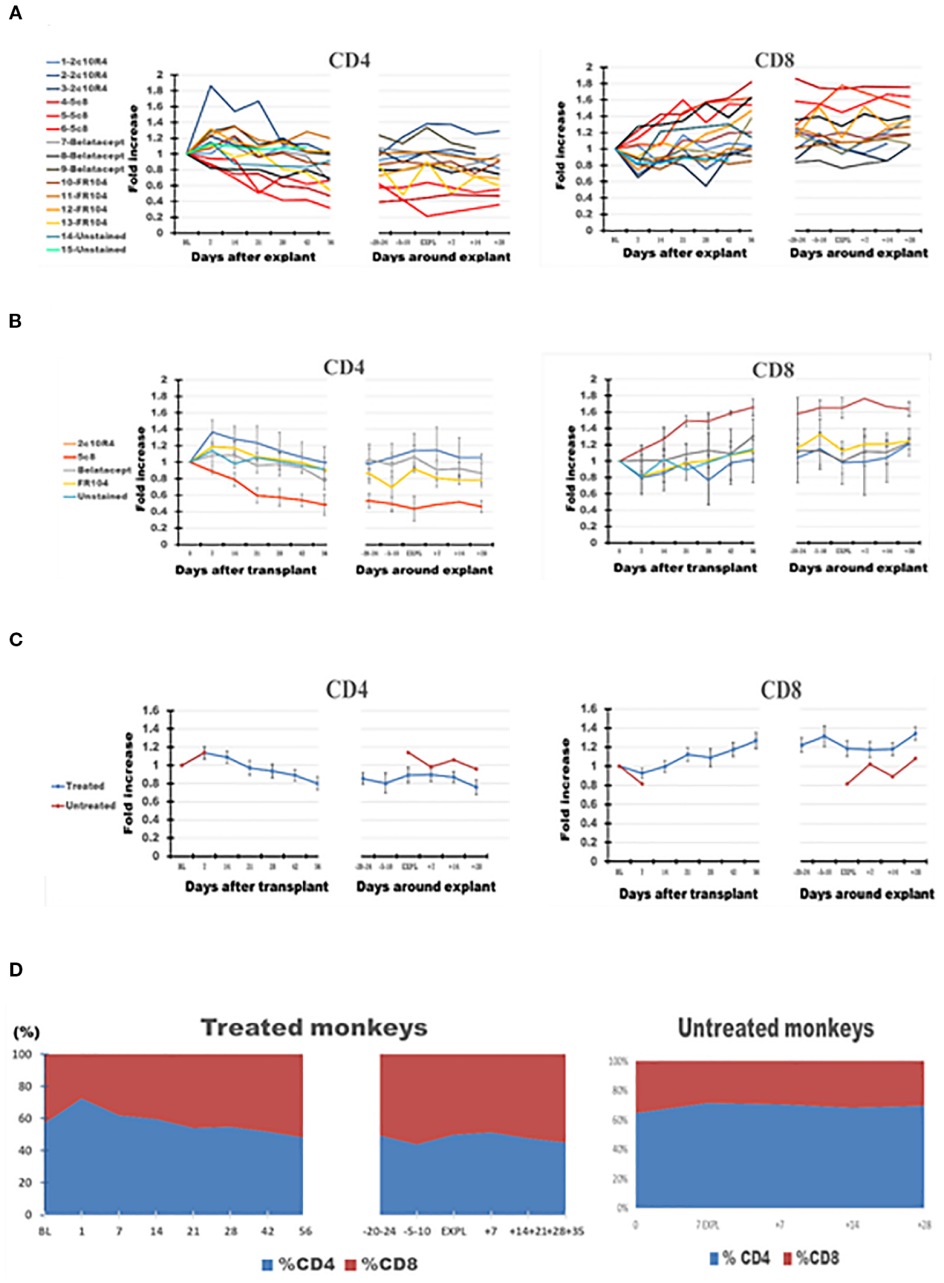
The dynamics of absolute CD4 and CD8 lymphocyte numbers in peripheral blood (PB) of the cynomolgus monkeys after a heterotopic (abdominal) heart transplantation. Absolute numbers of CD4+ T cells, and CD8 T cells, measured based on TruCount method results for lymphocytes (Ly) (as described in Materials and Methods section) as well as on T-cell subset percentages defined by flow cytometry (SI 3 and 4), are represented as fold changes versus values recorded before the transplant [baseline (BL) = mean values presented in **Table 3**]. For each graph, the measurements were represented post-transplant (left panel of each graph) or peri-rejection (right panel of each graph) at the indicated time points. **(A)** Results are shown for each monkey (n = 15) and color-coded as blue (2C10R4), red orange (5c8), gray (belatacept), yellow (FR104), and green spectrum (untreated). **(B)** Results are grouped by treatments, retaining color scheme from panel **(A)** Error bars reflect SEM. **(C)** Mean of all treated monkeys in association with stable graft function for all treatment groups (blue) versus untreated with acute rejection at D7 (orange). No significant differences from BL in aggregated data. However, CD4/CD8 ratio consistently trended down in all treated groups post-transplant and peri-rejection, most prominently in the 5c8 group, reflecting relative expansion of CD8+ T cells in circulation. The untreated group, during acute rejection at 7 days, presented a higher percentage of CD4 cells compared with CD8 cells. **(D)** Combined dynamic changes in the percentages of CD4+ cells (blue) and CD8+ cells (orange) in treated monkeys (left side of the panel) compared with untreated ones (right side of the panel).

PB CD4 cells decreased over time after transplant in most of the treated monkeys and were decreased at explant compared with BL (**Figure 4C**, panel 1), in contrast with a trend toward increasing numbers of CD8 cells (**Figure 4C**, panel 2). Consequently, on average, the CD4+/CD8+ ratio tended to decline in PB from 1.28 at BL to 1.05 at graft explant; a decline in CD4+/CD8+ ratio was also observed in mesenteric LNs between these two observation points from 3.11 to 2.87 (**Table 3**).

**Table 3:** The size of CD3 cell populations.

Cells	PB–BL	PB–EXP	GILs	LN–BL	LN–EXP
CD4	5,164 ± 221	4,773 ± 337	2,990 ± 227	7,263 ± 129	7,141 ± 286
CD8	4,008 ± 218	4,542 ± 380	5,414 ± 231	2,330 ± 148	2,489 ± 243
CD4+CD8+	478 ± 150	349 ± 67	1,392 ± 155	249 ± 54	254 ± 55
CD4−CD8−	289 ± 122	158 ± 28	41 ± 13	96 ± 28	79 ± 5
CD4 CD127lowCD25highFoxp3+	61 ± 17	84 ± 17	136 ± 27	471 ± 56	535 ± 73
CD8 CD127lowCD25highFoxp3+	6.4 ± 2.1	26 ± 9	97 ± 21	57 ± 33	106 ± 31
CD4+CD8+ CD127lowCD25highFoxp3+	12 ± 2.5	38 ± 11	220 ± 13	101 ± 23	161 ± 46
CD4−CD8− CD127lowCD25highFoxp3+	0	0	0.07 ± 0.07	1.1 ± 0.5	0.8 ± 0.3

The numbers of CD3+ cell populations (top four rows) and CD3+ CD127lowCD25highFoxp3+ cell subpopulations (bottom four rows) in peripheral blood (PB), graft-infiltrating lymphocytes (GILs) at explant, and graft-adjacent mesenteric lymph nodes (LNs). Categorized by the expression of CD4 and/or CD8. Results are expressed as means of all monkeys for the absolute numbers among 10,000 CD3+ cells obtained after flow cytometry analysis of each sample, as described in Materials and Methods.

### 3.4 CD127lowCD25highFoxp3+ CD3+ T cells in PB

Effective costimulation pathway-blocking treatments were associated with an increase in CD127lowCD25highFoxp3+ cells from 0.61% at BL to 0.84% at explant (1.3-fold) for CD4 cells (putative Treg CD4 cells), from 0.06% to 0.26% (4.06-fold) for CD8 cells, and from 0.12% to 0.38% (3.1-fold) for CD4+CD8+ cells (**Table 3**).

### 3.5 Comparison of GIL cell subpopulations

Based on the 39.6-g mean weight of rejected heart allografts and an average yield of 1 million GILs per gram of digested heart tissue, the total number of intragraft lymphocytes was estimated to be approximately 39.6 million. After correction for flow acquisition efficiency, doublets, and viability as described in SI 4, the rejected heart graft at explant contained an average of 12.5 million viable CD3 cells; of these, there were 3.73 million CD4 cells, 6.76 million CD8 cells, 1.74 million CD4+CD8+ cells, and 0.05 million CD4−CD8− cells, along with 5.94 million B cells. Quantitative estimates of cell numbers for cell subpopulations in GILs versus LNs or PB are detailed in SI-5.

**Figure 5:**
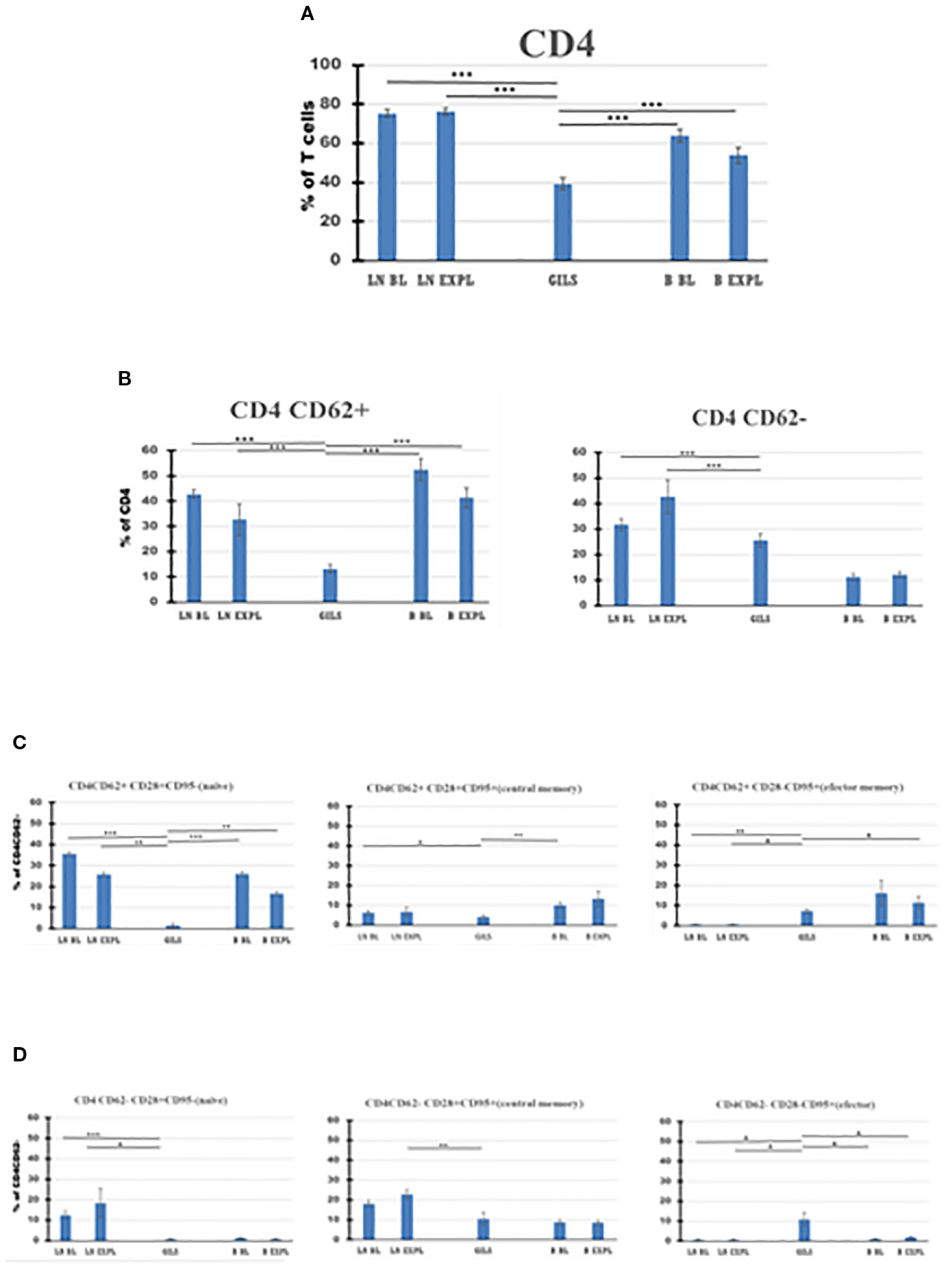
CD4 T-cell subpopulations in graft (GILs), compared with lymph nodes (LNs) and peripheral blood **(B)**, at baseline (BL) and around the time of explant (EXPL), after heterotopic heart transplantation. CD4 T-cell enumeration and gating strategies are presented in Materials and Methods section and detailed in SI 3 and 4. In graft at explant, the percentage of CD4 T cells was lower compared with peripheral blood (PB) or LNs (p < 0.001) **(A)**. The CD4 populations were further divided into CD62+ and CD62− subpopulations **(B)**. For both subpopulations, the percentage of cells was lower in GILs than in LNs (p < 0.001). Each of these two subpopulations was further analyzed using classical quadrant gate strategy for the expression of CD28 versus CD95 markers. For CD4 CD62+ cells in graft, a lower percentage of naïve cells compared with LNs (p < 0.01) or B (p < 0.01) (**C**, left graph) was noted. For CD4 CD62− cells in graft, an increase (p < 0.05) was noted for effector memory cells (**D**, right graph). For aggregated data, significant differences versus GILs are indicated for p < 0.05 (*), p < 0.01 (**), and p < 0.001 (***).

**Figure 6:**
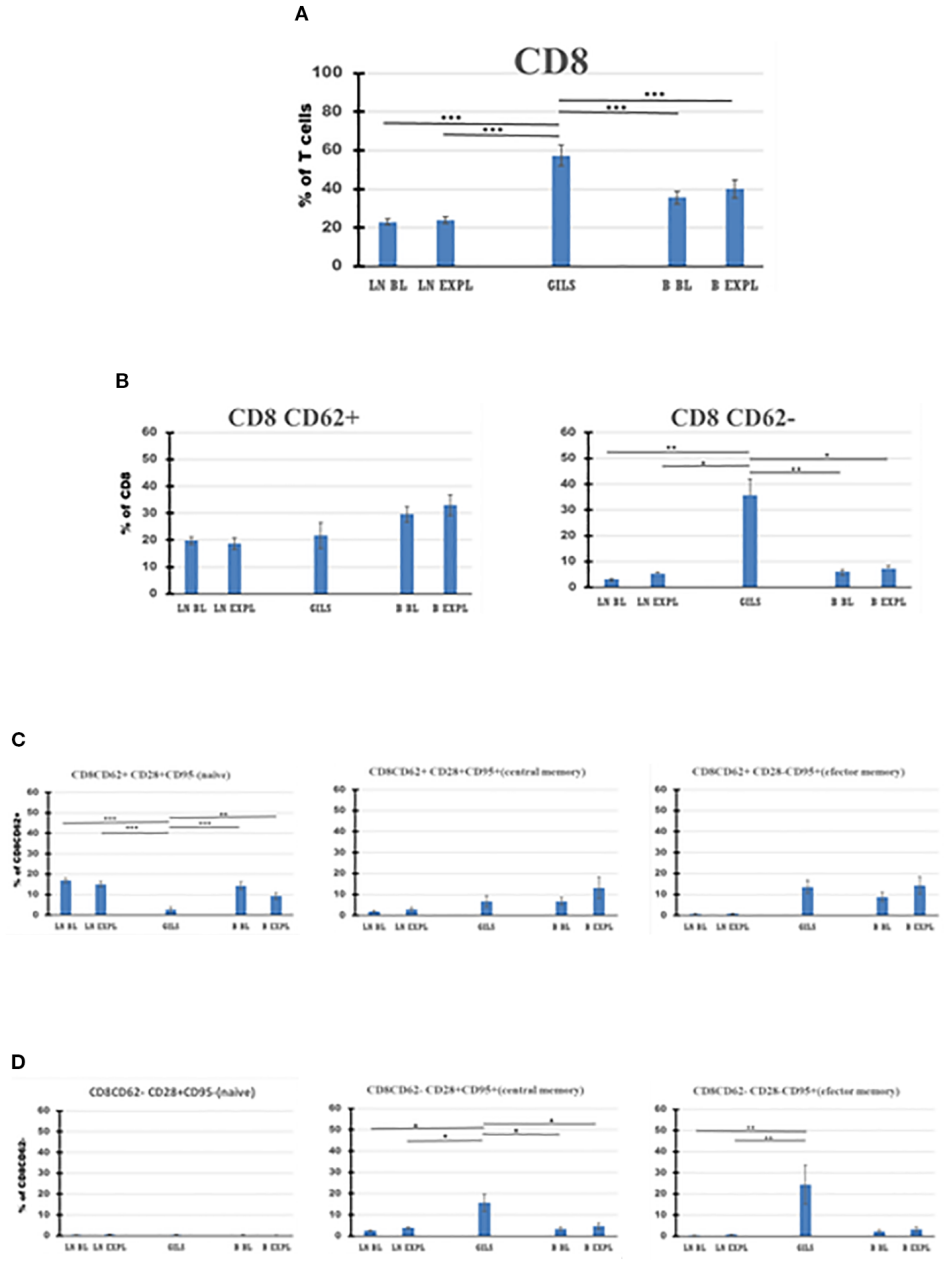
CD8 T-cell subpopulations in graft (GILs), compared with lymph nodes (LNs) and peripheral blood **(B)**, at baseline (BL) and around the time of explant (EXPL), after heterotopic heart transplantation. CD8 T-cell enumeration and gating strategies are presented in Materials and Methods section and detailed in SI 3 and 4. In graft at explant, the percentage of CD8 T cells is higher compared with peripheral blood (PB) or LNs (p < 0.001) **(A)**. The CD8 populations were further divided into CD62+ and CD62− subpopulations **(B)**. Between these, in GILs, an explant was detected with a higher percentage of CD8CD62− cells compared with LNs or PB (p < 0.05). Each of these two subpopulations was further analyzed using classical quadrant gate strategy for the expression of CD28 versus CD95 markers. For CD8 CD62+ cells in graft, a decrease (p < 0.01) of naïve cells (**C**, left graph) was noted. For CD8CD62− cells in graft, an increase (p < 0.05) was noted for central memory cells (**D**, middle graph). For aggregated data, significant differences versus GILs are indicated for p < 0.05 (*), p < 0.01 (**), and p < 0.001 (***).

In the graft at the time of rejection, in contrast to PB or LNs, CD8 cells comprised the highest percentage of CD3 cells (p < 0.001) and the lowest CD4 cells (p < 0.001) (**Figures 4A–D**, **5A–D**, **6A–D**, **Table 3**). Consequently, the CD4/CD8 ratio in GILs was 0.5 in association with graft rejection, compared to 1.29 in PB or 2.86 in LNs at that time point. CD4+CD8+ DP cells represented 13.92% of CD3+ GILs at explant, fourfold higher than in blood (3.49%) and 5.5-fold higher than in LNs (2.54%) at the same time point (**Table 3**).

Based on the surface expression of CD62 on CD4 or CD8 subclasses of CD3 cells (**Figures 5**, **6B**), the percentage of activated CD8+CD62− cells was higher in the graft at explant compared with LNs (p < 0.05) or PB (p < 0.05) (**Figure 6B**, panel 2), and the quiescent CD4 CD62+ cell percentage was smaller in GILs than in LNs (p < 0.001) or blood (p < 0.001) (**Figure 5B**, panel 1). GILs contained a smaller percentage of CD4 and CD8 CD62+ naïve T cells (T_N_) (CD28+CD95−) relative to LNs (p < 0.001) or blood (p < 0.01) (**Figures 5**, **6C**, panel 1). In contrast, the percentage of both CD4 and CD8 CD62− effector memory T cells (T_EM_) (CD28−CD95+) (**Figures 5**, **6D**, panel 3) and CD8+ central memory cells (T_CM_) (CD62−CD28+CD95+) T cells (**Figure 6D**, panel 2) increased in GILs (p < 0.05) compared with PB or LNs.

Among CD127lowCD25highFoxp3+ cells in the rejected graft, 30% were CD4+, 21% were CD8, and 49% were CD4+CD8+; the ratio between CD4 and CD8 single-positive putative Treg among GILs was 1.4. The percentage of CD4 CD127lowCD25highFoxp3+, putative CD4 Treg cells (1.36% ± 0.27% from CD3 cells) in GILs, was increased compared with that in blood (2.2-fold higher at BL and 1.6-fold higher at explant), but it was lower compared with that in LNs (3.5-fold lower than LNs at BL and 3.9-fold lower at explant) (**Table 3**).

Putative Treg CD8 cells (CD127lowCD25highFoxp3+) were enriched in GILs compared with PB (15-fold compared with PB at BL and 3.7-fold versus PB at explant) or 1.7-fold higher than LNs at BL, but were around the same proportion as in LNs at explant (1.1 ratio) (**Table 3**). The ratio of CD4/CD4 Treg cells in GILs was 22, compared with a ratio of 56 for CD8/CD8 Treg cells.

Comparison of cluster analysis of CD3 CD127lowCD25highFoxp3+ cell subpopulations (CD4, CD8, and DP) between GILs, LNs, and PB (14 clusters; **Table 4**) identified a significant increase in the number of two clusters of DP cells and of three clusters in CD8 in GILs. For instance, cluster 2 of DP, putative regulatory cells, in GILs included 82 cells, compared with PB (8 cells) or LNs (6 cells) (p < 0.001). In cluster 3, the differences were smaller: 17 cells in GILs, compared with 3 in PB and 1 in LNs (p < 0.05). Likewise, the number of CD8+ CD127lowCD25highFoxp3+ cells increased in GILs in clusters 1, 3, and 5 compared with PB or LNs. An increase in cell numbers in GILS was noted for cluster 2 of CD8 cells in hu5c8-treated monkeys (**Table 4** in bold font). Cluster 4 was enriched with DP cells in LN and GILS compared with PB (p < 0.001) (**Table 4**). Clusters 4 and 8 for CD4+Cd127lowCD25highFoxp3+ cells were enriched in LN compared with GILS or PB (p < 0.001) (**Table 4**).

**Table 4:** Cluster analysis of CD3 CD127lowCD25highFoxp3+ cells subpopulations.

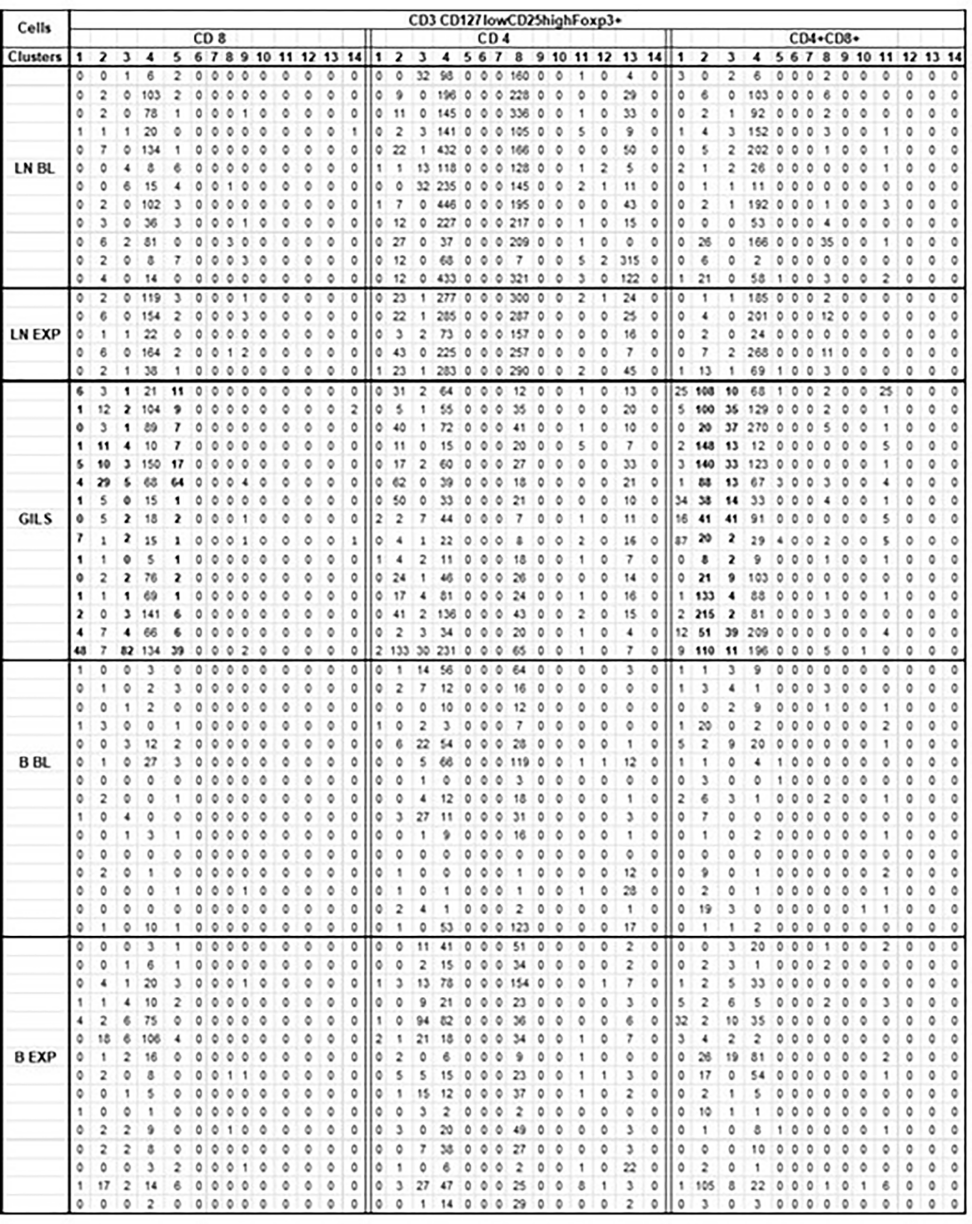

For each monkey (rows) for each corresponding sample, flow cytometry results for CD3 population were downsized to same number of 10 000 cells as described (SI-4 step 5). For further analysis of the 62 unmerged files (No samples obtained for 3 BL and 10 EXP LN) it was used SOM tool of FCSExpress 7. Each CD127lowCD25highFoxp3+ cells subpopulation (CD8, CD4, CD4+CD8+) were divided into 14 clusters (columns) as illustrated.

For CD4+CD8+ CD127lowCD25highFoxp3+ cells in GILS it was observed a significant difference (p<0.001), regarding the size of cluster 2 (bold) that included a mean of 82 cells, compared with PB where the same cluster consists of only 8 cells, or LN that included 6 cells. In cluster 3 (bold) the differences were smaller (17 cells in GILS, compared with 3 in PB and 1 in LN) but still significant (p<0.05). Likewise, for CD8+CD127lowCD25highFoxp3+ cells, the number in GILS (bold) was increased (p<0.01) in clusters 1, 3, and 5 compared with PB or LNs.

### 3.6 B cells

**Figure 7:**
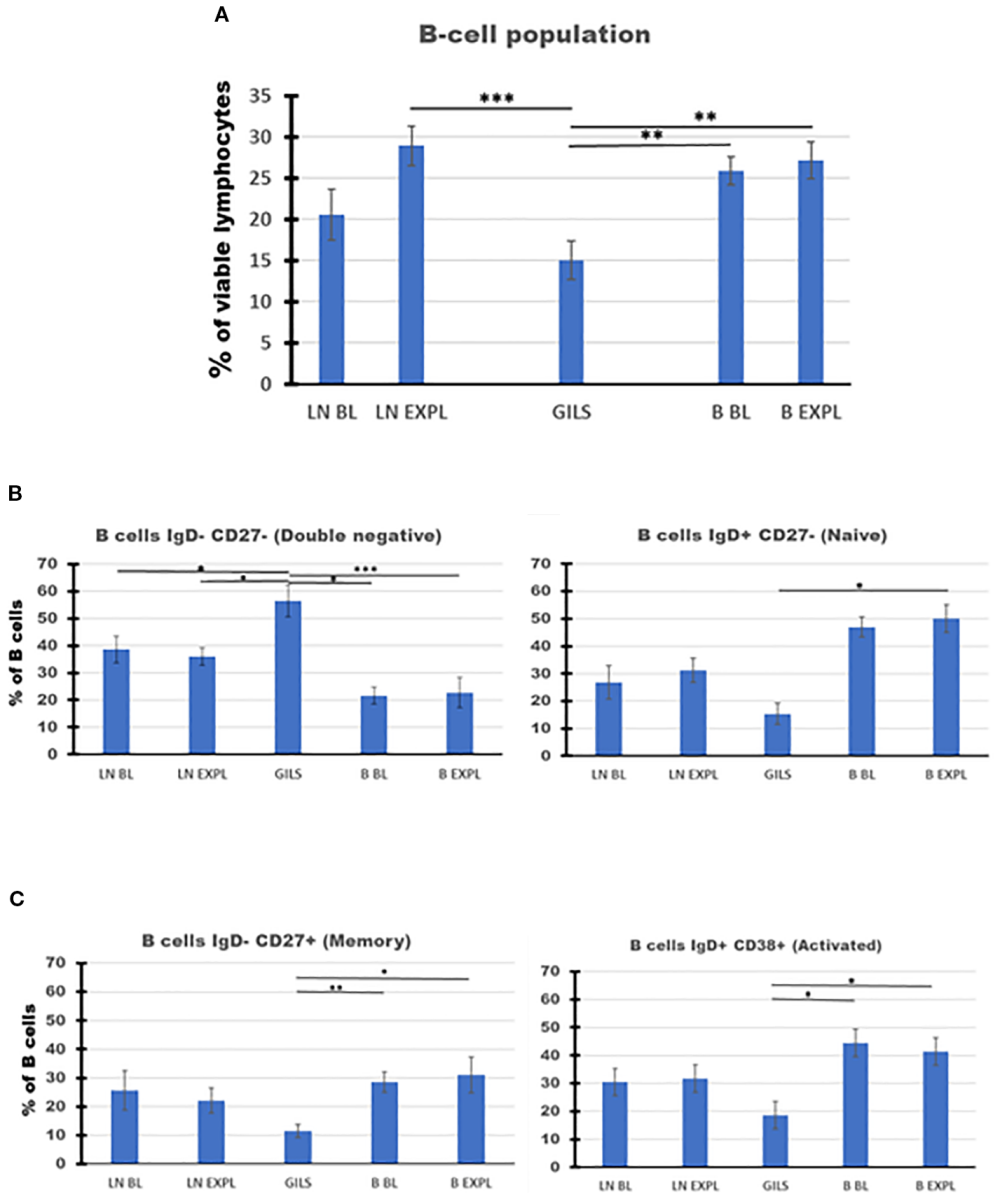
B-cell subpopulations in lymph nodes (LNs), rejected grafts (GILs), and peripheral blood (PB) after heterotopic abdominal heart transplantation. B cells, as described in SI 4 (II Gating strategy for B cells) and using BOLEAN gate CD19orCD20, are represented as mean of all treated heart allograft recipients (n = 13), for the percentage from viable lymphocytes (Ly) **(A)**. B-cell percentage at explant was lower in graft compared with LNs (p < 0.001) or blood (p < 0.01). The B-cell populations were further analyzed using classical quadrant gate strategy for expression of IgD versus CD27 and IgD versus CD38 markers. The results are expressed as a percentage of 1,000 B cells obtained after the use of weighted density downsizing sampling tool of FCS Express-7. **(B, C)** Subpopulations presented significant differences between compartments. In the graft at explant, there were noted decreased percentages for naïve, memory, and activated B cells, compared with peripheral blood (PB) (p < 0.05). In contrast, a significant increase in GILs for the percentage of double-negative B cells was recorded compared with LNs (p < 0.05) or PB (p < 0.01). For aggregated data, significant differences versus GILs are indicated for p < 0.05 (*), p < 0.01 (**), and p < 0.001 (***).

The decrease in PB B cells associated with the transplant episode rebounded more slowly than did T cells (**Figure 2C**, panels 1 and 3). At the time of explant, the percentage of B cells in GILs was low compared with PB (p < 0.01) or LNs at explant (p < 0.001) (**Figure 7**, panel A). The percentage of double-negative IgD−CD27− B cells was higher in GILs compared with PB (p < 0.01) or LNs (p < 0.05) (**Figure 7B**, panel 1), whereas the percentage of naïve, memory, or activated B cells was lower among GILs compared with LNs or PB (**Figure 7B**, panel 2, **Figure 7C**).

## 4 Discussion

The presented dynamics of various leukocyte subpopulations after transplantation in PB and LNs, compared with values in heart allograft during rejection, reflect the immune system’s complex coordinated responses (7) to continuous exposure to graft antigens in the context of IS treatments and the proinflammatory stress induced by the surgical procedure. Our findings align with conventional allograft rejection paradigms and provide new relevant data. Comparison of the results between the three compartments analyzed (PB, LNs, and GILs) in this pilot study yielded several testable working hypotheses regarding differences between IS regimens with mechanistic and therapeutic implications.

To the extent that PB is viewed simplistically as a readily accessible biologic transport medium for leukocytes, the significant increase of WBC after transplantation and peri-rejection represents the cumulative result of their constitutive population dynamics. Changes described for different leukocyte populations, during the first 56 days of stable graft function and around the time of explant, generally show a similar pattern between monkeys across treatment groups, albeit with differences in amplitude associated with treatments, especially prominent for belatacept and hu5c8.

Our results suggest a prominent role of innate immune cells for heart allograft rejection in non-human primates (NHPs) treated with costimulation pathway blockade. First, after an initial transient increase in the number of neutrophils post-transplantation driven by stress response to surgery (8), another neutrophil peak appeared around the time of graft rejection and explant. This pattern appeared to be blunted in the belatacept group; we are aware of no costimulation mechanism-based explanation for this observation. Second, monocytes, which are mobilized into PB under circumstances of systemic stress (9) and play a critical role in tissue development, homeostasis, and injury repair (10), also expanded in the PB post-transplant and then again peri-explant. We speculate that these innate immune surveillance cells are mobilized by cytokines elaborated in response to the systemic stress associated with transplant surgery (11) and with local inflammation during graft rejection. The detection of a second increase in the number of PB monocytes around the time of rejection implicates this population in graft injury and deserves further study, especially given the emerging role of non-classical monocytes in alloantigen recognition and immunity (12). Currently, elevated M numbers in PB have been proposed as a biomarker predictive of acute kidney injury outside the transplant context (13) and associated with clinical kidney transplant rejection (14). Increased monocyte-to-neutrophil ratio was associated with cardiac arrhythmic complications after heart transplant (15), and the lymphocyte-to-monocyte and neutrophil-to-monocyte ratios were proposed to quantify the inflammatory profile in hyperlipidemic patients undergoing PCSK9 inhibitor treatment (16). Third, our results draw particular attention to eosinophils, which consistently underwent a relative expansion in peripheral blood in anticipation of and in association with graft rejection, particularly in the belatacept group. Eosinophils are implicated in the pathogenesis of a variety of immune-mediated diseases (17) such as allergies, asthma (18), pemphigoid, systemic sclerosis, and sarcoidosis; in various hypereosinophilic syndromes characterized by eosinophil-mediated organ damage (19, 20); and in the Th2 response to parasitic infections (21). They are also associated with post-transplant organ homeostasis (22) as well as graft rejection following heart (23), lung (24), kidney (25), and liver (26) transplantation. The heart is one of the preferential targets for eosinophil inflammation in up to half of the patients with hypereosinophilic syndrome (19). The reason for their preferential homing in the myocardium during systemic inflammation is unknown (17). In fact, 0.5% of myocardial autopsies show signs of eosinophil infiltration regardless of the inciting cause (27). One clinical study also suggested that monitoring peripheral blood E counts may be useful to predict early acute heart allograft rejection (23). Their involvement in immunological mechanisms of graft rejection may include their under-appreciated role as antigen-presenting cells (28, 29), and their cytokine secretion profile may in turn influence T-cell proliferation, monocyte/macrophage activation, and B-cell antibody production (30, 31). Recently, eosinophils have been implicated in the induction of donor HLA-specific IgE antibody after sensitization by heart and skin grafts in mice and human kidney transplant recipients (32). Further, since the expression of CD40 ligand (CD154) on activated E binding to CD40 constitutively expressed on endothelial cells has been implicated in certain chronic inflammatory conditions (33, 34), blockade of the CD40/CD154 pathway would be expected to attenuate graft injury to the extent that it is mediated by CD154-expressing E. These studies, coupled with our presented observations, suggest that monitoring of E numbers, perhaps in association with anti-donor IgE levels, may be useful to enhance the sensitivity of rejection surveillance strategies designed to minimize dependence on protocol graft biopsies. It is difficult to determine if observed eosinophilia contributes to graft rejection or is a result of different rejection mechanisms. However, considering their complex roles in immune response, the increase in E number after transplantation, especially before rejection, may be of significance. Considering observed variations in the eosinophil numbers induced by different treatments, it is tempting to speculate that, like in asthma where only 60% of adult patients have elevated levels of E (35), eosinophilia may be an effective mechanism of rejection in a subset of transplanted patients. After further validation, it may be helpful to consider that, in addition to targeting cytokines, the patients with allotransplants who present hypereosinophilia may benefit from treatments that directly target the E number as it increases.

According to the transplant immunology canon, lymphocytes are the principal vectors of the adaptive immune response. It is tempting to speculate that the decline of Ly in PB on the first day after transplant reflects their net migration into the graft, graft-draining lymph nodes, and spleen, as it was observed in both treated and untreated animals. Classically, donor antigens presented by APCs to host T cells (by direct, indirect, or semidirect mechanisms) after ligation with T-cell receptors trigger “signal 1” for T-cell activation. Reciprocal T cell–APC activation in turn also stimulates the upregulation of the endothelial venule expression of CCR7 and other molecules that may favor lymphocyte homing to germinal centers. In the context of costimulation pathway blockade that prevents “signal 2”, reducing pathogenic alloimmunity and, to variable degrees depending on the pathway, facilitating the emergence of graft-protective peripheral immunoregulation, it was observed that Ly counts rebound to or above BL. The rise of Ly counts in PB in anticipation of graft failure, particularly in the hu5c8 and belatacept groups, where treatment was stopped after D84, may reflect expansion of the circulating donor-specific lymphocyte pool. This increase was not seen in association with electively explanted beating grafts in two 2C10R4-treated animals still under medication at D84. We speculate that the subsequent decrease in PB Ly around the time of explant reflects the sequestration of expanded donor-reactive Ly subpopulations migrating into the graft, presumably en route to mediate allograft rejection. CD3 T-cell and B-cell subpopulations follow a similar dynamic as Ly, presenting lower numbers in PB after the transplant procedure. However, CD3 T cells rebound to baseline by approximately 7 days after transplant, whereas B cells take approximately 40 days to recover. Further analysis of CD3 T-cell subpopulations in PB identified a steady increase in the proportion of CD8 cells relative to CD4, with changes in the CD4/CD8 ratio like those observed in a pig-to-NHP islet xenotransplantation model (36).

Lymph nodes, especially those near the graft, are key sites where both donor antigen presentation to T cells and subsequent proliferation of donor-specific lymphocytes are presumed to occur. Consistent with the classical transplant immunology model, we observed a relative expansion of CD4+CD62− T cells in LNs at the time of rejection compared with BL, presumably predominantly a “helper” phenotype, and a decrease in the population of CD4+CD62+ naïve cells. The population of CD8+ T cells in LNs at the time of rejection tends to be numerically higher, likely representing the primary source of pathogenic donor-specific effectors that migrate to the graft. Overall, our observations are in keeping with the classical working model that CD8 cells migrating from peripheral blood into the allograft are the primary effectors inducing cellular allograft injury. The increased percentage of B cells in LNs at explant may illustrate their increasing role with time to present donor antigen to T cells and to generate anti-donor antibodies.

It was presumed that the T-cell subtypes in the transplant may mediate graft fate since the graft is the logical location to measure the balance between rejection effector and graft-protective immunomodulatory mechanisms. Rejecting grafts exhibited a smaller percentage of CD4 cells and a higher percentage of CD8 cells relative to PB or LNs, findings that are concordant with previous descriptions of cell-mediated allograft rejection in rodent models (37) and clinical studies (38). Loss of expression of adhesion molecule CD62L is associated with cellular activation (39) during Ly homing to LNs or in inflammation (40). The higher proportion of central (CD28+CD95+) and effector memory (CD28−CD95+) cells found among CD8+CD62− T cells at the level of the graft further reinforces the described primary role of activated effector CD8 cells in local rejection mechanisms. Comparing Ly in the graft versus PB at explant, the graft was relatively enriched for CD127lowCD25highFoxp3+ cells among CD4, CD8, and CD4+CD8 cells by 1.6-, 3.7-, and 5.7-fold, respectively, and by 2.2-, 15-, and 18-fold versus PB at BL, respectively. Surprisingly, double-positive CD4+CD8+ (DP) T cells constituted approximately 13.9% of CD3+ cells in rejected grafts, compared with 3.5% in blood and 2.5% in LNs, suggesting preferential homing or intragraft expansion. DP lymphocytes, presumably immature, are not normally found in substantial numbers outside of the thymus. Their increased number in peripheral blood has been associated with several pathological conditions, including cancer, autoimmune disorders, and viral infections, and with graft rejection in a non-human primate model of islet transplantation (41). The significance of a high percentage of DP T cells among GILs is not known. So far, single-cell RNA sequencing in a cynomolgus monkey model has demonstrated heterogeneity of this cell population (42). Further characterization of CD127lowCD25highFoxp3+ cell subpopulations among the CD8, CD4, and double-positive GIL populations, in comparison with PB or LNs after transplant, has the potential to enhance our understanding of graft rejection mechanisms or to offer new therapeutic targets to reach tolerance. B cells are known to be involved in antibody response and also in antibody-independent functions such as modulation of T-cell differentiation, promoting T-cell survival through antigen presentation, the regulation of macrophage phenotype, and the production of both regulatory (anti-inflammatory and proinflammatory) cytokines. The low percentage of B cells in the graft at the time of rejection compared with the blood or LNs deserves further investigation. The role of a high proportion of double-negative (IgD−CD27−) B cells in GILs, also observed in patients with autoimmune (43) and infectious diseases, including SARS-CoV-2 (44), remains speculative. The lower percentages of naïve, memory, and activated B cells in GILs may be of significance.

The discovery of new leukocyte subpopulations will lead to the development of new approaches for diagnosing or combating rejection (45, 46). It is expected that the identification of putative new subsets of cells with specific properties may herald rejection or indicate the need for treatment changes to maintain tolerance. More granular definition of their phenotype and function may yield new insights into innate and adaptive immune rejection mechanisms (47–51). Our presented cluster analysis may be an example in this direction. In the future, the use of advanced flow cytometry methods (52–54), including new cell sorting strategies (55–57), together with modern “omics” techniques (58), will enhance the detection limit of differences at the single-cell level (59, 60). Continuation of the reductive approach to the molecular level may also suggest new hypotheses or lead to the discovery of new complex biomarkers (61) currently under investigation, such as cell-free DNA (62), microRNA (63), or glycoproteins (64).

The present study has limitations of a descriptive retrospective “pilot” work. First, the small number of cases with a complete set of data for each costimulation blockade regimen (partly due to the complexity of the experimental model or logistic constraints) describes an exploratory study that may suggest further developments. The accuracy, reproducibility, and mechanistic significance of the presented observations will require further strategically targeted studies. Second, the GIL analyses characterized a single time point during advanced stages of graft rejection, which may not illustrate other, and potentially mechanistically important, cell populations present in the graft at earlier time points, which may better inform strategies to prevent graft injury. Further studies need to be conducted to address this question. Finally, the relevance to humans of NHP-derived data remains to be established, particularly in relation to the demonstration of clinical efficacy for blockade of different costimulation pathways and for the evaluation of graft rejection mechanisms.

In conclusion, peripheral blood leukocyte populations revealed similar patterns of modulation across the various costimulation pathway-blocking regimens used in this study and in association with subsequent graft rejection after treatment cessation. Expansion of CD8+ T cells in peripheral blood in anticipation of graft failure and their predominance in rejected grafts support the conventional paradigm regarding the role of activated CD8 T cells as key effector cells mediating graft injury. Our data demonstrate a previously undescribed expansion of circulating eosinophils around the time of rejection, supporting the testable hypothesis that preventing eosinophil expansion, or interfering with their function, may address a costimulation pathway-resistant mechanism of allograft injury. The prominence of CD4+CD8+ (DP) T and IgD−CD27− (DN) B lymphocytes within the rejecting graft also implicates these cell subpopulations in pathogenic alloimmune responses, a hypothesis that deserves further investigation. Changes in T Foxp3+ subpopulations in GILs compared with PB, especially the increase in the numbers of cells in specific clusters at the time of rejection, may suggest the important roles of these cells in revealing the immune mechanisms of rejection. Based on this pilot survey, we conclude that future efforts to elucidate the roles of specific PB, LN, and GIL cell subpopulations in larger groups of animals treated with consistent regimens are a promising strategy to identify new candidate approaches to prevent allograft rejection and promote tolerance.

## Data Availability

The raw data supporting the conclusions of this article will be made available by the authors, without undue reservation.
