# Correction: Leukocyte dynamics in *Cynomolgus* monkeys following heterotopic heart allotransplantation under costimulation pathway blockade

**DOI:** 10.3389/fimmu.2025.1732897

**Published:** 2025-12-03

**Authors:** Gheorghe Braileanu, Agnes M. Azimzadeh, Tianshu Zhang, Lars Burdorf, Richard N. Pierson

**Affiliations:** 1 Department of Surgery, University of Maryland Medical Center, Baltimore, MD, United States; 2 Division of Cardiac Surgery, Department of Surgery and Center for Transplantation Science, Massachusetts General Hospital and Harvard Medical School, Boston, MA, United States

**Keywords:** leucocyte dynamics, cynomolgus macaque, heart heterotopic allotransplantation, intragraft lymphocytes, T cells subpopulation

There was a mistake in **Table 4** as published. The misplacement of horizontal lines in column 1 changes repartition of events in each compartment making it impossible to substantiate interpretations of data from the results section. In addition, the published PDF table is spread on 3 pages instead of one (as it was initially submitted or as it appears on my projects site); it should be on one page. Besides, in published PDF format the horizontal and vertical lines specifically indicated in Q11 are not bold, making it very difficult to read the table. The corrected **Table 4** appears below.

**Table 4:** Cluster analysis of CD3 CD127lowCD25highFoxp3+ cells subpopulations.

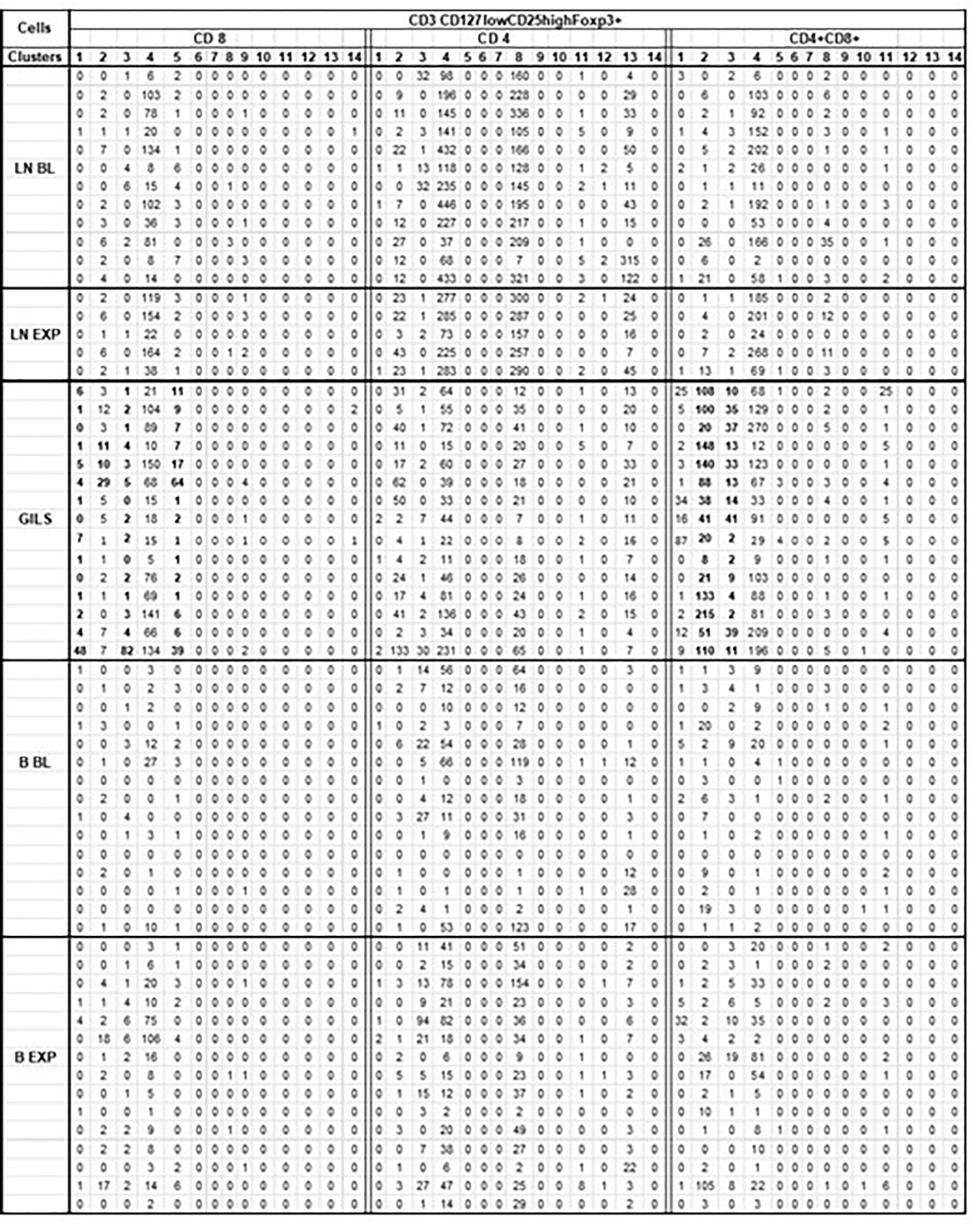

For each monkey (rows) for each corresponding sample, flow cytometry results for CD3 population were downsized to same number of 10 000 cells as described (SI-4 step 5). For further analysis of the 62 unmerged files (No samples obtained for 3 BL and 10 EXP LN) it was used SOM tool of FCSExpress 7. Each CD127lowCD25highFoxp3+ cells subpopulation (CD8, CD4, CD4+CD8+) were divided into 14 clusters (columns) as illustrated.

For CD4+CD8+ CD127lowCD25highFoxp3+ cells in GILS it was observed a significant difference (p<0.001), regarding the size of cluster 2 (bold) that included a mean of 82 cells, compared with PB where the same cluster consists of only 8 cells, or LN that included 6 cells. In cluster 3 (bold) the differences were smaller (17 cells in GILS, compared with 3 in PB and 1 in LN) but still significant (p<0.05). Likewise, for CD8+CD127lowCD25highFoxp3+ cells, the number in GILS (bold) was increased (p<0.01) in clusters 1, 3, and 5 compared with PB or LNs.

Also, there was a mistake in the caption of **Table 4** as published. Some numbers are wrong: …5-replace with 8; eight-replace with 6; 1 replace with 3; 2.7-replace with 1; Some abbreviations were misplaced: … blood-replace with PB; add “PB or” in front of LNs.

“For each monkey (rows) for each corresponding sample, flow cytometry results for CD3 population were downsized to same number of 10–000 cells as described (SI-4 step 5). For further analysis of the 62 unmerged files (No samples obtained for 3 BL and 10 EXP LN) it was used SOM tool of FCSExpress 7. Each CD127 lowCD25 highFoxp3+ cells subpopulation (CD8, CD4, CD4+CD8+) were divided into 14 clusters (columns) as illustrated.

For CD4+CD8+ CD127lowCD25highFoxp3+ cells in GILS it was observed a significant difference (p<0.001), regarding the size of cluster 2 (bold) that included a mean of 82 cells, compared with PB where the same cluster consists of only 5 cells, or LN that included 8 cells. In cluster 3 (bold) the differences were smaller (17 cells in GILS, compared with 1 in blood and 2.7 in LN) but still significant (p<0.05). Likewise, for CD8+CD127lowCD25highFoxp3+ cells, the number in GILS (bold) was increased (p<0.01) in clusters 1, 3, and 5 compared with LNs.”

The corrected caption of **Table 4** appears below.

“For each monkey (rows) for each corresponding sample, flow cytometry results for CD3 population were downsized to same number of 10–000 cells as described (SI-4 step 5). For further analysis of the 62 unmerged files (No samples obtained for 3 BL and 10 EXP LN) it was used SOM tool of FCSExpress 7. Each CD127lowCD25highFoxp3+ cells subpopulation (CD8, CD4, CD4+CD8+) were divided into 14 clusters (columns) as illustrated.

For CD4+CD8+ CD127lowCD25highFoxp3+ cells in GILS it was observed a significant difference (p<0.001), regarding the size of cluster 2 (bold) that included a mean of 82 cells, compared with PB where the same cluster consists of only 8 cells, or LN that included 6 cells. In cluster 3 (bold) the differences were smaller (17 cells in GILS, compared with 3 in PB and 1 in LN) but still significant (p<0.05). Likewise, for CD8+CD127lowCD25highFoxp3+ cells, the number in GILS (bold) was increased (p<0.01) in clusters 1, 3, and 5 compared with PB or LNs.”

The original version of this article has been updated.

