# Supplementary material for: Leukocyte dynamics in Cynomolgus monkeys following heterotopic heart allotransplantation under costimulation pathway blockade
Source: Front Immunol. 2025 Oct 10;16:1664463. doi: 10.3389/fimmu.2025.1664463 (PMC12549273; doi:10.3389/fimmu.2025.1664463)
Supplement: Supplementary file 1 [file DataSheet1.zip › SI 1/SI - 1.docx]

**SI - 1**

**Monitoring of allotransplanted hearts; criteria for explant**

Selection of donor-recipient pairs was based upon compatible blood group and major histocompatibility complex incompatibility as gauged by stimulatory index in mixed lymphocyte reaction > 5 (not shown), as previously reported(1). Graft survival was monitored by an implanted telemetry device (D70-PCTP, Data Science International, St. Paul, MN). Core temperature and graft heart rate, systolic blood pressure and diastolic blood pressure were assessed at least once daily using intra-abdominal telemetry until time of graft explant. Grafts were explanted when contractility deteriorated, based on the criteria of a pulse pressure <30 mmHg for 2 consecutive days, or a drop-in heart rate to <120 beats/min or a decline of >40 beats/min from a stable baseline. Graft dysfunction was confirmed by palpation, ultrasound and direct visual confirmation before explant (2).

1. Azimzadeh AM, Zhang T, Wu G, Kelishadi SS, Stoddard T, O'Neill N, et al. Preemptive CD20+ B cell Depletion Attenuates Cardiac Allograft Vasculopathy in CD154-Treated Monkeys. Transplantation. 2017;101(1):63-73.

2. Zhang T, Azimzadeh AM, Sun W, O'Neill NA, Sievert E, Bergbower E, et al. Selective CD28 Inhibition Modulates Alloimmunity and Cardiac Allograft Vasculopathy in Anti-CD154-Treated Monkeys. Transplantation. 2018;102(3):e90-e100.
