# Supplementary material for: Leukocyte dynamics in Cynomolgus monkeys following heterotopic heart allotransplantation under costimulation pathway blockade
Source: Front Immunol. 2025 Oct 10;16:1664463. doi: 10.3389/fimmu.2025.1664463 (PMC12549273; doi:10.3389/fimmu.2025.1664463)
Supplement: Supplementary file 2 [file DataSheet2.zip › SI 2/SI - 2.docx]

**SI - 2**

**Protocol for isolation of intragraft lymphocytes (GILS)**

1) Weigh the part of the graft used for cell extraction as well as the whole heart.

2) Mince the graft piece, up 8 g, with a scissor, in a Petri dish with 10 ml Gibco 1640 RPMI medium (Thermo-Fisher Scientific, Hampton, NH, USA) to yield 1-2 mm^3^ pieces.

3) Centrifuge heart fragments in a 50 ml tube (1400 rpm for 5 min at 4 ^o^C) and discard the supernatant.

4) Resuspend the pellet with 24 ml of collagenase solution prepared as follow:

1g collagenase Type 4 (Worthington Biochemical Corporation Lakewood, NJ, USA)

100 mg DNase I, from bovine pancreas grade II (Boehringer Mannheim Corp, Roche, cat. nr. 10104159001, Millipore Sigma, St. Louis, MO)

1g Soybean Trypsin Inhibitor (cat nr. T9128, Millipore Sigma, St. Louis, MO)

Dissolve ingredients in 1 L 50:50 DMEM:F12 (Gibco+L glutamine+15 mM HEPES), filter, sterilize.

Aliquot and store at 20 C.

5) Incubate resuspended heart fragments for 30 min in a water bath at 37 C for 30 min, briefly agitating the tube every 10 min.

6) Pass the digested heart fragments through a metal mesh and add RPMI with 10% NBCS to inactivate enzymes in a Petri dish.

7) Centrifuge dissociated cells in two 50 ml tubes (1400 rpm for 5 min at 4 ^o^C), discard the supernatant, resuspend both pellets in 40 ml media.

8) Add 20 ml of heart cells suspension to each of two new 50 ml tubes. With a 10 ml pipet add 10 ml FICOLL paque (GE Healthcare, NY, USA) at the bottom of each tube.

9) Centrifuge at 2800 rpm, for 20 min, no brake, at room temperature.

10) Harvest the leukocyte buffy coat at the Ficol-media interface.

11) Wash leukocyte buffy coat with complete media (RPMI with 10% FCS and antibiotics: P+S)

12) Resuspend buffy coat in 5 ml of complete media, count, and process for further analysis.
