# Supplementary material for: Leukocyte dynamics in Cynomolgus monkeys following heterotopic heart allotransplantation under costimulation pathway blockade
Source: Front Immunol. 2025 Oct 10;16:1664463. doi: 10.3389/fimmu.2025.1664463 (PMC12549273; doi:10.3389/fimmu.2025.1664463)
Supplement: Supplementary file 3 [file DataSheet3.zip › SI 3/SI - 3.docx]

**SI – 3**

**Flow cytometry material and methods**

The flow cytometer (FACSVerse – BD Bioscience, San Jose, CA, USA) equipped with 3 lasers that can read 8 colors, from which the APC-Cy7 channel was reserved for testing cell viability by e780 blue dye exclusion.

**3.1. Enumeration of leukocytes**

Enumeration of leukocytes in peripheral blood at each protocol-driven timepoint and on the day of graft explant was done using three independent methods. The absolute number of total white blood cells (WBCs) and their populations were determined by flow cytometry using TRUCOUNT tubes (BD Bioscience, San Jose, CA, USA), deploying the formula provided by the manufacturer (SI – 3.1.1 below). Leukocytes were also independently enumerated using 2 automated hemocytometers: on an in-house Hemavet 950 (Drew Scientific Group, Miami Lakes, FL, USA) following the manufacturer protocol; and by ANTECH Diagnostics (LA, USA). WBC results were usually concordant between the three methods (r=0.9685). In rare instances (< 2%), when results from one method were discrepant, the value supported by the other two measurements was reported; otherwise, the TruCount flow results were used.

To reduce the influence of variability of BL values between monkeys and focus on intra treatment groups-level, and analyzed population-level changes over the peri-transplant and peri-rejection intervals, results are expressed as fold change (increase or decrease) in respect to the value for that individual monkey at BL. The absolute numbers of circulating CD3+ T cells, NK and B cells were additionally assessed based on the absolute TruCount numbers and the percentages of each of these subpopulations obtained from the flow cytometry results analysis.

**3.1.1.** **TruCount Procedure**

1. Add all antibodies to each TruCount tube, just above the retainer.

2. Add 50 μl of well mixed (by inversions) blood on EDTA to each tube (Use reverse pipetting)

3. Vortex briefly and incubate 15 min. at room temperature (RT) in dark.

4. Add 450 μl of BDLyse (349202) to each tube. Do not vortex.

5. Incubate 15 min. at room temperature in the dark.

6. Vortex and acquire by flow cytometry using prior settings.

| **Antibody** | **Label** | **Clone** | **Source** | **Volume per sample (μl)** |
| --- | --- | --- | --- | --- |
| CD3 | V450 | SP34-2 | BD (Horizon) | 4 |
| CD2 | FITC | RPA-2.10 | BD | 5 |
| CD20 | PE | 2H7 | BD (Pharmingen) | 5 |
| CD45 | PerCP | D058-1283 | BD | 5 |

Absolute count of the cell population (A) = Number of positive cell events (X) divided by the number of bead events (Y), and then multiplying by the BD TruCount bead concentration (N/V, where N is number of beads per test, and V is testing volume) A=X/YxN/V

**3.2. Protocols – direct surface stain**

**3.2.1. Blood**

1. Prepare a stock of antibodies cocktail for a corresponding panel, adjusted with FC-PBS to use 50 μl peer tube. Add 50 μl blood per sample. Mix.

2. 30 min on ice.

4. For blood, add 2 ml 1xRBC Lysis buffer (BD cat 00-4333) to each tube and mix well - 12 min at RT.

5. Spin 1500 rpm x 5 min and decant the supernatant.

6. Wash cells with 2 ml FC-PBS and spin 1500x5min.

7. Add 300 μl PFA-FIX – Vortex – wait 30 min at RT.

8. Wash cells with 2 ml FC-PBS and spin 1500x5min.

9. Add 300 μl PBS/tube, vortex and acquire by flow cytometry using prior settings.

**3.2.2. LN and GILS**

1. Add 5x10^5^ cels/tube.in 40 μl in FC-PBS, 5 μl hIgG and 5 μl of e780 (1/42 in FSB) per tube. (The hIgG and e780 are added to all cell suspensions before aliquoting in tubes).

2. 15 min on ice.

3. Add corresponding cocktail of surface antibodies adjusted with FC-PBS to use 50 ml per tube. Mix.

4. 30 min on ice.

Continue with step 6 from above

| **Panel (T)** | **V450** | **V500** | **FITC/Alexa488** | **PE** | **APC** | **PeCy7** | **PerCP** | **APC-Cy7** |
| --- | --- | --- | --- | --- | --- | --- | --- | --- |
| 1 | IgG1.k | IgG1.k | IgG | IgG1 | IgG2a | IgG1 | CD8 | e780 |
| 2 | CD3 | CD4 | CD45RA | CD62L | CD95 | CD28 | CD8 | e780 |

| **Panel (B)** | **V450** | **V500** | **FITC/Alexa488** | **PE** | **APC** | **PeCy7** | **PerCP** | **APC-Cy7** |
| --- | --- | --- | --- | --- | --- | --- | --- | --- |
| 1 | IgG1,k | IgG | IgG1,k | IgG1 | CD19 | IgG1 | CD20 | e780 |
| 2 | CD21 | CD27 | Goat a-IgD | CD38 | CD19 | IgM | CD20 | e780 |
| 3 | CD3 | CD4 | CD45RA | CD62L | CD95 | CD28 | CD8 | e780 |

|  |  |  |  |  |  |  |  |  |
| --- | --- | --- | --- | --- | --- | --- | --- | --- |
| **Antibody** | **Label** | **Clone** | **Source** | **Vol (μl/sample)** |  |  |  |  |
| IgG1,k | V450 | MOCP-21 | BD Horizon | 5 |  |  |  |  |
| CD21 | V450 | B-ly4 | BD Horizon | 2 |  |  |  |  |
| CD3 | V450 | SP34-2 | BD Horizon | 4 |  |  |  |  |
| IgG | V500 | X40 | BD Horizon | 5 |  |  |  |  |
| CD27 | V500 | M-T271 | BD Horizon | 2.5 |  |  |  |  |
| CD4 | V500 | L200 | BD Horizon | 1 |  |  |  |  |
| IgG | FITC | MOPC-21 | Pharmingen | 10 |  |  |  |  |
| CD45RA | FITC | 5H9 | Pharmingen | 4 |  |  |  |  |
| a-IgD | FITC | Polyclonal | Southern-Biotech | 0.5 |  |  |  |  |
| IgG2a.k | PE | H129.19 | Pharmingen | 20 |  |  |  |  |
| CD62L | PE | SK11 | Pharmingen | 0.5 |  |  |  |  |
| CD38 | PE | OKT10 | NHPR Resource | 1 |  |  |  |  |
| IgG | APC | MOPC-21 | Pharmingen | 20 |  |  |  |  |
| CD95 | APC | Dx2 | Pharmingen | 4 |  |  |  |  |
| CD19 | APC | J3-119 | Beckman-IOTest | 10 |  |  |  |  |
| IgG | PeCy7 | MOPC-21 | Pharmingen | 5 |  |  |  |  |
| CD28 | PeCy7 | CD28.2 | BD Pharmingen | 2.5 |  |  |  |  |
| IgM | PeCy7 | 1B4B1 | Southern-Biotech | 1 |  |  |  |  |
| CD8 | PerCP | SK1 | Bio Legend | 5 |  |  |  |  |
| CD20 | PerCP | L27 | BD | 1 |  |  |  |  |

**3.3. Protocol - intracellular staining for Foxp3**

1. Use 100 μl blood per tube. LN or GILS cells were suspended in 95 μl of Flow Staining Buffer (FSB)

(BD cat 00-422) supplemented with 5 μl hIgG.

2. Add 5 μl of e780 (1/42 in FSB) and keep on ice for 15 min.

3. Add surface antibodies - 30 min on ice.

4. For blood, add 2 ml 1xRBC Lysis buffer (BD cat 00-4333) to each tube and mix well - 10 min at RT.

5. Spin 1500 rpm x 5 min and decant the supernatant.

6. Wash cells with 2 ml FSB and 1500x5min.

7. Add 1 ml of fresh Foxp3 Fixation/permeabilization solution (eBioscience)/sample. Pulse vortex. Place

for 2.5 h on ice, in dark, or overnight

8. Wash the blood cells with 2 ml FSB, or 2 ml of 1x Permeabilization (PERM) buffer #3 (eBioscience)

for LN or GILS. Spin 1500 rpm x 5 min.

9. Block blood pellet w 2 μl normal mouse serum in 90 μl FSB. For LN or GILS use 90 μl of 1x PERM

buffer #3. On ice for 15 min

10.Without washing add Foxp3 or isotype control antibody (in FSB for blood or PERM buffer #3 for

LN or GILS) fill with FSB or PERM buffer #3 ad 100 μl. Keep on ice and dark for 30 min.

11.Wash with 2 ml FSB for blood or 1xPERM buffer #3 for LN or GILS. Spin 1500 rpm x 5 min and

decant the supernatant.

12.Resuspend in 300 ml PBS and acquire or fix the cells to run next day.

| **Panel (Foxp3)** | **V450** | **V500** | **FITC/Alexa488** | **PE** | **APC/AlexaF647** | **PeCy7** | **PerCP** | **APC-Cy7** |
| --- | --- | --- | --- | --- | --- | --- | --- | --- |
| 1 | CD3 | CD4 | IgG | Hst IgG | IgG1,k | IgG | CD8 | e780 |
| 2 | CD3 | CD4 | CD25 | Rbt IgG | IgG1,k | CD127 | CD8 | e780 |
| 3 | CD3 | CD4 | CD25 | IgG2a.k | Foxp3 | CD127 | CD8 | e780 |
| 4 | CD3 | CD4 | CD25 | CD122 | Foxp3 | CD127 | CD8 | e780 |

|  |  |  |  |  |  |  |  |  |
| --- | --- | --- | --- | --- | --- | --- | --- | --- |
| **Antibody** | **Label** | **Clone** | **Source** | **Vol (μl/sample)** |  |  |  |  |
| CD3 | V450 | SP34-2 | BD Horizon | 4 |  |  |  |  |
| CD4 | V500 | L200 | BD Horizon | 1 |  |  |  |  |
| IgG | BB515 | X40 | BD Horizon | 5 |  |  |  |  |
| CD25 | BB515 | 2A3 | BD | 5 |  |  |  |  |
| Hamster IgG | PE | NKT888 | Bio Legend | 2 |  |  |  |  |
| Rabbit IgG | PE | A95-1 | BD Pharmingen | 5 |  |  |  |  |
| IgG2a.k | PE | H129.19 | Pharmingen | 20 |  |  |  |  |
| CD122 | PE | Mik-beta2 | BD Pharmingen | 20 |  |  |  |  |
| IgG1k | APC/AlexaF647 | P3.6.2.8.1 | eBioscience | 1 |  |  |  |  |
| Foxp3 | APC/AlexaF647 | 236A/E7 | eBioscience | 5 |  |  |  |  |
| IgG | PeCy7 | MOPC-21 | Pharmingen | 5 |  |  |  |  |
| CD127 | PeCy7 | hIL-7R-M21 | BD Pharmingen | 5 |  |  |  |  |
| CD8 | PerCP | SK1 | Bio Legend | 5 |  |  |  |  |
