# Supplementary material for: Leukocyte dynamics in Cynomolgus monkeys following heterotopic heart allotransplantation under costimulation pathway blockade
Source: Front Immunol. 2025 Oct 10;16:1664463. doi: 10.3389/fimmu.2025.1664463 (PMC12549273; doi:10.3389/fimmu.2025.1664463)
Supplement: Supplementary file 4 [file DataSheet4.zip › SI 4/SI - 4.docx]

**SI – 4**

**Analysis of flow cytometry results**

**Gating for TRUCOUNT leucocytes numbers analysis**


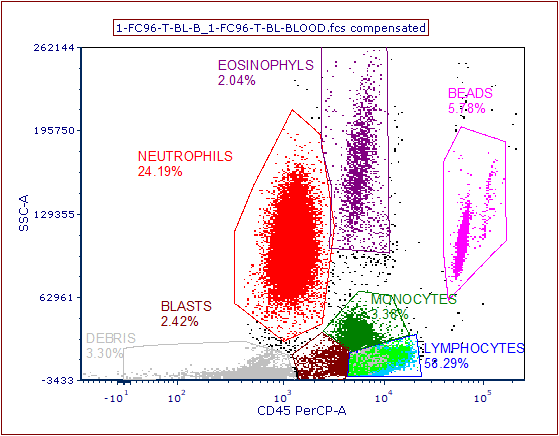


**Strategy for lymphocytes analysis**

The acquired files for lymphocytes identification were analyzed with FCSExpress7 software (De Novo Software Pasadena, CA, USA) using the next steps:

1. Cleaning of the total number of acquired events. FlowAI predefined algorithm of FCS express cleaning tool
2. Exclusion of the doublets FCS-A/FCS-W and SCS-A/SCS-W gates
3. Selection of the viable cells Exclusion dye e789 (eBioscience)
4. Gating CD3 positive cells anti CD3 monoclonal antibody
5. Downsizing CD3 population to 10 000 events Down sampling tool of FCS express
6. Apply classical gating strategy
7. For cluster analysis, 62 files (Blood at BL and explant and GILS at 15 files each, and existing LN files) at 10 000 CD3 cells each were first merged to obtain meaningful results. Using flow SOM in FCS express, the cluster analysis was applied to each unmerged file by k-means methodology for 14 centroids. Cells in the same group (i.e. cluster) are more similar each other than those in other groups (i.e. other clusters).


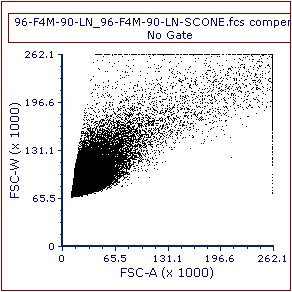
^
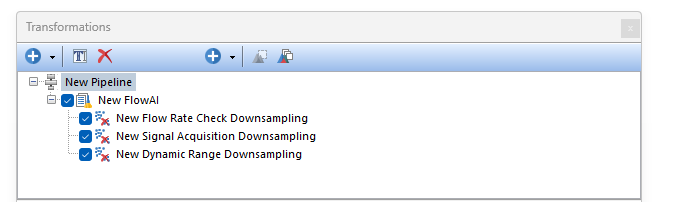
^
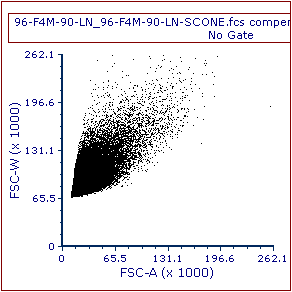


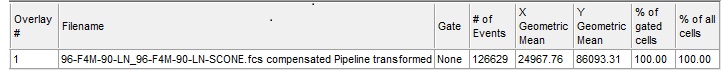

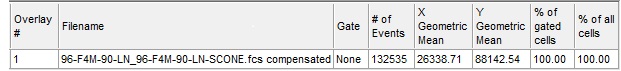


1. Cleaning of FCS files using FlowAI tool of FCSExpress 7.


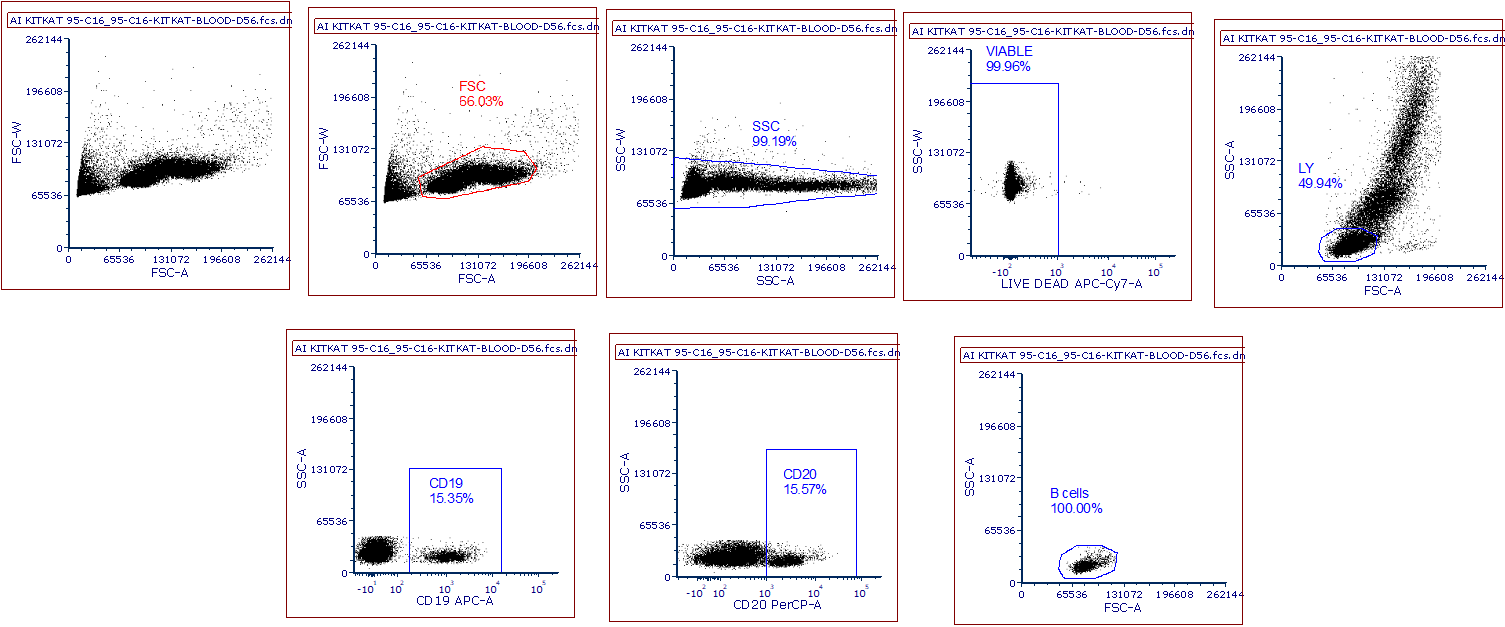


1. Gating strategy for B cells.

FCS files were first cleaned using AI tool of FCSExpress 7. B cells population was obtained using the Boolean gate CD19orCD20. For further analysis of B cell subpopulations between compartments (PB, graft, LN) the initial number of B cells for each sample was first equalized to 1 000 using the SOM tool of FCSExpress7.


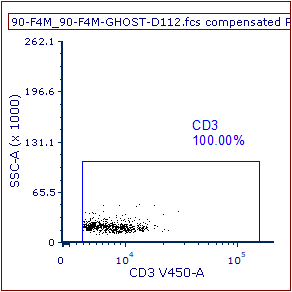

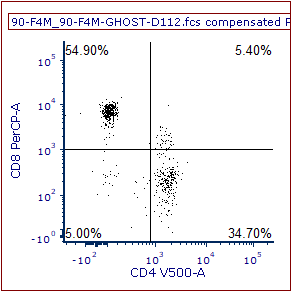

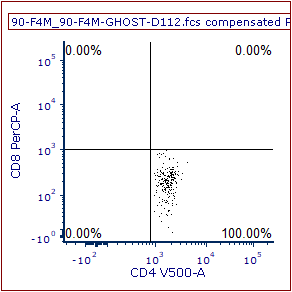

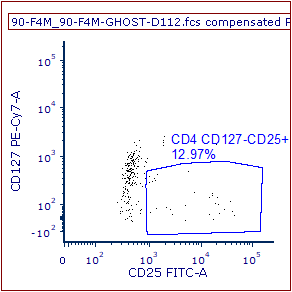

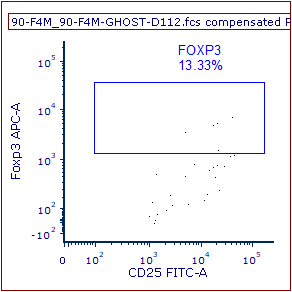


1. Classical gating strategy for CD4 CD127lowCD25highFoxp3+ cells.

For each sample the initial number of CD3 cells was first downsized to 10 000 cels using SOM tool of FCSExpress7.
