# Supplementary material for: Leukocyte dynamics in Cynomolgus monkeys following heterotopic heart allotransplantation under costimulation pathway blockade
Source: Front Immunol. 2025 Oct 10;16:1664463. doi: 10.3389/fimmu.2025.1664463 (PMC12549273; doi:10.3389/fimmu.2025.1664463)
Supplement: Supplementary file 5 [file DataSheet5.zip › SI 5/SI - 5.docx]

SI – 5

**Quantitative considerations**

Estimation of total body numbers of leucocytes in PB, and of lymphocytes subpopulations in heart graft and LN, in Cynomolgus monkey.

**Blood**

Based on an estimated total blood volume of 590 ml (8% (1) from 7.373 kg medium body weight of experimental Cynomolgus monkey’s), TruCount results and the percentages of gated subpopulations, the absolute numbers of leucocytes at D0, were around 6.577 billion, of which 3.164 billion neutrophils, 2.704 billion lymphocytes, 575 million monocytes, and 152 million eosinophils. Of the lymphocytes, 1922 million were CD3, from which 992 million were CD4, 769 million CD8, 91.8 million CD4+CD8+, and 55.5 million CD4-CD8- cells. The ratio between CD4 and CD8 cells was 1.28. Regarding CD3CD127lowCD25highFoxp3 cells in PB calculated for BL averaged 26/μl, of which about 20 cells/μl were CD4+, 2 cells/μl CD8+ and 4 cells/μl CD4+CD8+. Consequently, their total numbers were 11.8 million for CD 4, 1.18 million for CD8 and 2.36 million for DP. There were a total of 656 million B cells.

**GILS**

Based on the 39.6 g mean weight of rejected heart allografts and an average yield of 1 million GILS per gram of digested heart tissue, the total number of GILS is around 39.6 million. From these, an average of about 169,000 CD4CD127 lowCD25highFoxp3+ cells, 121,250 CD8CD127lowCD25highFoxp3+ cells and 245,000 CD4+CD8+CD127 lowCD25highFoxp3+ cells were present in the heart at rejection. In GILS, the ratio between CD4 and CD8 cells was 0.55, the ratio between CD4 CD127 lowCD25highFoxp3+ versus CD8 CD127lowCD25highFoxp3+ cells was 1.4.

**LN (without spleen, bone marrow, or tissue resident lymphocytes)**

The total number of lymph nodes in a Cynomolgus monkey was estimated by our surgeons to be 125. From a monkey mesenteric LN weighing 0.15 g were obtained a mean of 4 million cells. If all lymph nodes average this size, it means that a monkey has a total of 500 million lymphocytes resident in LN. After corrections for singlets and viability, a monkey’s LN compartment contains 214 million CD3 cells at BL and 212 million at explant. From these, 155 respectively 152 million are CD4, 49.9 respectively 53 million are CD8, 5.3 respectively 5.4 million are CD4+CD8+ cells, and between 2.1 respectively 1.7 million are CD4-CD8- cells. The ratio between CD4 and CD8 cells was 3.1 at BL and 2.87 at explant. The number of CD127lowCD25 highFoxp3+ T cells were between 10.1 respectively 11.4 million cells for CD4, 1.2 respectively 2.2 million cells for CD8, 2.2 respectively 3.4 million for CD4+CD8+ cells, and 0.02 respectively 0.016 million for CD4-CD8- cells (meaning 6.48% respectively 7.49% from CD4, and 2.44% respectively 4.25% from CD8). The ratio between CD4 and CD8 T of these cells was 8.32 at BL and 5.07 at explant.

With possible practical value, presented numbers may suggest the order of magnitude for changes needed to be induced by eventual future targeted cellular interventions. For instance, a constant supply of millions of naive or transformed cells presumptively have the potential to change the balance for some specific subpopulations in size of hundreds of thousands of cells in the graft, that may be important for tolerance.

1. Hobbs TR, Blue SW, Park BS, Greisel JJ, Conn PM, Pau FK. Measurement of Blood Volume in Adult Rhesus Macaques (Macaca mulatta). J Am Assoc Lab Anim Sci. 2015;54(6):687-93.
